# Lived experience preferences for mental health peer support services in Australian primary care: a discrete choice experiment with qualitative analysis

**DOI:** 10.1136/bmjopen-2025-112132

**Published:** 2026-11-09

**Authors:** Billingsley Kaambwa, Belinda Fuss, Taylor-Jade Woods, Megan Rattray, Tania Shelby-James, Christine Kaine, Sharon Lawn

**Affiliations:** 1 Flinders Health and Medical Research Institute, Flinders University, Adelaide, South Australia, Australia; 2 College of Medicine and Public Health, Flinders University, Adelaide, South Australia, Australia; 3 College of Human Sciences and Culture, Flinders University, Adelaide, South Australia, Australia; 4 Lived Experience Australia, Adelaide, South Australia, Australia

**Keywords:** MENTAL HEALTH, Primary Health Care, Patient Preference, HEALTH ECONOMICS, Patient-Centered Care

## Abstract

**Objectives:**

To understand the preferences of Australian peer support workers (PSWs) and peer service recipients (PSRs) with lived experience towards the design of a mental health peer support service in primary care.

**Design:**

An online discrete choice experiment (DCE) asking respondents to indicate their preferences between two mental health peer support services in primary care based on eight attributes.

**Setting:**

Australia.

**Participants:**

A national panel of mental health PSWs and PSRs with diverse lived experience backgrounds.

**Main outcome measures:**

DCE respondent preferences regarding PSW qualifications, PSW experience, location of peer support sessions, integration with primary care, duration of programme, PSW’s identity/diagnosis/cultural background, frequency of sessions and format of peer support. Attitudinal responses and contextualising comments for inductive reflexive thematic analysis were also collected.

**Results:**

84 respondents completed the DCE. Respondents were predominantly female (73%) and middle-aged (45–64 years, 62%) and most PSWs had paid experience in peer work (93%). The parsimonious random-parameter logit model indicated preferences for community-based or home-based delivery, weekly, biweekly or on-demand sessions, individual delivery, formally qualified and experienced PSWs and longer programme durations. Pooled interaction analyses found no statistically significant overall differences in preferences between PSWs and PSRs. The opt-out alternative was selected in 50 of 672 choice tasks (7.4%). Themes identified from comments included: (1) frustration with the DCE format; (2) the medical model and peer work; (3) identifying with multiple roles; and (4) contextualising preferences.

**Conclusions:**

This DCE provides evidence on respondents’ preferences for the design and delivery of mental health peer support programmes in primary care. Overall, respondents prefer frequent, community-based or home-based individual peer support delivered by qualified, experienced PSWs. The findings provide directions for establishing acceptable peer support services in primary care.

**STRENGTHS AND LIMITATIONS OF THIS STUDY**

- We used a discrete choice experiment to provide evidence for mental health peer support work (PSW), giving nuanced insights into service delivery features preferred by respondents.
- Thematic analysis of contextualising comments enriched quantitative findings, capturing complex views of peer support in mental health.
- Purposeful sampling ensured representation of PSWs and peer service recipients, valuing professional expertise and lived experience.
- Role categorisation (‘peer worker’ vs ‘recipient’) may have oversimplified respondent identities, as some drew from both their PSW and lived experiences.
- The targeted, small sample limits generalisability to the general population.

## Introduction

 Shared lived experiences between a formal ‘peer support worker’ (PSW) and a peer service recipient (PSR), also referred to as a ‘consumer’ in the Australian context, form the backbone of peer support services. As an integral component of mental healthcare, peer support services involve leveraging shared lived experiences to promote recovery and resilience.^1^ In this paper, PSWs refer to individuals employed or engaged in designated lived experience roles who intentionally use their lived experience of mental health challenges, recovery and/or caring for someone experiencing mental ill health to support others. This differs from ‘informal peer support’, which occurs naturally between people with shared experiences outside a designated service role. We use the term ‘PSR’ to describe individuals receiving support from a formal PSW in a mental health or primary care setting.^1^ By sharing their own recovery journeys, PSWs create an environment of trust and mutual understanding, building relationships centred on equality and reciprocity.^2 3^ In doing so, PSWs reduce stigma and foster hope in individuals navigating similar challenges.^4^ This is a unique form of support that is intentionally distinct from traditional mental health interventions in moving away from an overly medicalised model of care.^5^ By addressing emotional, practical, and social needs, peer support services provide a person-centred and recovery-oriented approach that is increasingly promoted as a best-practice alternative to more clinician-driven approaches.^6 7^

Peer support services are widely acknowledged to bridge critical gaps in conventional mental healthcare systems. These gaps often include accessibility barriers, a lack of culturally appropriate services and limited personalisation in care delivery.^8 9^ By offering services rooted in lived experience, PSWs help individuals feel heard and validated, providing a sense of community that traditional services sometimes fail to provide. Research suggests that peer support services can improve mental health outcomes, including reduced hospital readmissions, improved quality of life and increased engagement in care.^10 11^

Additionally, there is a growing need to integrate mental health services with primary care across Australia, as mental health is most commonly accessed through general practitioners (GPs).^12^ Attending the GP for mental health needs, however, does not necessarily lead to a formal diagnosis or referral to a secondary care provider such as a psychologist, psychiatrist, community mental health clinical team or non-clinical psychosocial support service. PSWs and the support that they provide have been considered as one form of support at this level. Despite this, there is little available research into best practice for how this should be approached within the primary care context.

Despite the documented benefits of peer support,^13–16^ significant knowledge gaps remain in understanding how best to design and deliver peer support services to meet the diverse needs of PSRs. The heterogeneity among individuals who access these services, including differences in demographic, mental health conditions, and cultural contexts, necessitates a tailored approach to service design. For instance, attributes such as the qualifications and experience of PSWs, and the format of sessions (eg, group vs one-on-one) may significantly influence PSR preferences and engagement.^1 16 17^ These attributes also fit within the general preferences of PSRs in primary care, where attributes of the care provider, wait time between appointments, duration of care and shared decision-making were among the most important attributes.^18^

To address similar gaps in knowledge, researchers are increasingly turning to discrete choice experiments (DCEs), a robust methodology rooted in health economics and decision sciences. DCEs are particularly well-suited to explore preferences for complex service attributes by presenting individuals with hypothetical scenarios composed of varying combinations of attributes and levels.^19^ Respondents are asked to select their preferred option, allowing researchers to quantify the relative importance of each attribute and examine trade-offs individuals are willing to make. This method is widely used in healthcare research to inform patient-centred care models in primary care,^18^ telehealth,^20^ palliative care^21^ and chronic disease management.^22^

In the context of peer support services, a DCE offers a powerful tool to identify the specific attributes that matter most to both PSWs and PSRs. For example, prior research has highlighted the importance of flexibility in service delivery, the need for community-based options and the value of experienced PSWs.^7 8^ However, these studies often rely on qualitative or survey-based methods, which lack the precision and granularity provided by a DCE. By examining preferences for key attributes such as session format, provider qualifications, and integration with clinical services, a DCE can offer actionable insights for policymakers and service providers seeking to optimise peer support programmes.^1 16 17^

Moreover, understanding preference heterogeneity is critical to addressing equity in mental healthcare. Different population subgroups, such as individuals from culturally and linguistically diverse backgrounds or those living in rural and remote areas, may prioritise attributes differently. For example, rural residents may value community-based services that minimise travel, while culturally diverse populations may prioritise PSWs with similar lived experiences or cultural understanding.^23 24^ DCEs enable the exploration of such heterogeneity, offering a nuanced understanding of how to tailor services for diverse user groups.^22^

This study builds on existing literature by employing a DCE to examine preferences for peer support service attributes among PSWs and PSRs. By capturing the trade-offs that individuals are willing to make, the study aims to provide evidence-based recommendations for designing effective, user-centred peer support programmes. The findings contribute to the growing body of research on lived experience in mental healthcare, offering critical insights for enhancing the accessibility, equity and effectiveness of peer support services.

## Methods

### Discrete choice experiment

DCE is a stated preference quantitative technique originating in mathematical psychology which is designed to establish the relative importance and impact of individual attributes, or characteristics, on the overall utility of a good or service.^25^ In a DCE, respondents are presented with a sequence of hypothetical scenarios (choice sets) made up of two or more competing alternatives that vary according to attributes that define them.^26^ For each choice set, respondents choose their preferred scenario that, based on the Lancasterian framework,^27^ represents the alternative with the highest utility.^26^ From these responses, the probability of choosing an alternative as a function of the attributes and other factors can then be calculated allowing this method to go beyond the traditional qualitative assessments and provide quantifiable data depicting the strength of respondents’ preferences for particular attributes.^26^ Unlike traditional ranking and rating exercises, DCE also goes further to provide quantitative information on marginal rates of substitution across attributes, trade-offs between attributes and predicted probabilities of uptake or demand for particular alternatives.^26^ Another advantage of DCE generated preferences for alternative scenarios is the ability to apply them within the framework of economic evaluation to inform healthcare management decision-making.^28^ This DCE was reported in accordance with the Design and Reporting of Discrete Choice Experiments in Healthcare (DIRECT) Checklist,^29^ available in the online supplemental material.

### Attribute development

The development of DCE attributes (specific features of peer support services that respondents traded-off when making choices) for this study followed established best-practice guidelines.^30 31^ Attributes were developed iteratively through a rigorous process that combined evidence synthesis with stakeholder co-design.

The evidence synthesis drew primarily on three reviews considered most relevant to the objectives of this DCE: a narrative review of peer support in mental health services,^7^ a systematic review and meta-analysis of peer support interventions for people with severe mental illness,^32^ and a rapid review and environmental scan of peer workforce models, implementation challenges and innovative service delivery approaches commissioned by the Australian Government Department of Health.^33^ Together, these reviews synthesised evidence on the defining characteristics of peer support, PSW qualifications and experience, models of service delivery, and implementation within mental health, primary care and community settings. The project lead had previously led the Australian Government rapid review of peer workforce models and implementation,^33^ and this work provided an important evidence base for identifying candidate attributes relevant to contemporary peer support services.

Drawing on these evidence sources, an initial list of 16 candidate attributes was identified. The preliminary attribute list was subsequently refined through iterative discussions with the multidisciplinary research team and a steering group comprising PSRs with lived experience of mental health challenges, PSWs, family carers, service providers and primary care researchers. Candidate attributes were considered according to their relevance to the study objectives, alignment with the core principles of peer support, policy and practice importance, feasibility for inclusion in a DCE, comprehensibility for respondents, potential overlap with other attributes and respondent burden. Potential conceptual overlap between candidate attributes was also considered during refinement to minimise redundancy while ensuring that the principal dimensions of peer support service delivery were represented. This iterative process resulted in the selection of eight attributes representing meaningful and actionable variations in peer support service delivery. Online supplemental table S1 presents the initial 16 candidate attributes, the primary evidence sources informing each attribute, and the rationale for their retention, modification, or exclusion.

The final attributes and levels with simplified descriptions were:

1. PSW qualifications: peer qualification, obtaining qualification or lived experience only.
2. PSW experience: less than 6 months, 6–12 months, 12–24 months or more than 24 months.
3. Location of peer support sessions: primary care clinic, community, online or respondent’s home.
4. Peer support integration with primary care: offered within the primary care clinic, external services, established contact persons or a combination of in-clinic and external options.
5. Duration of peer support programme: 4 weeks, 8 weeks, 12 weeks or ongoing/open-ended.
6. Identity, diagnosis, and cultural background of peer support provider: similar age, similar diagnosis, similar gender or similar culture.
7. Frequency of peer support sessions: monthly, biweekly, weekly or on-demand.
8. Format of peer support: online chat/forum, phone/video calls, individual sessions or group sessions.

### Survey development and pilot testing

The DCE survey was structured into three sections to optimise respondent engagement and data quality:

Section A: Descriptive statistics

This section collected demographic and background information, including age, gender and prior experience with peer support services.

Section B: Attitudinal questions

Respondents were asked to rate their agreement with 11 statements on the value and delivery of peer support services using a Likert scale. These statements acted as a warm-up to familiarise respondents with the key themes of the survey.

Section C: DCE

The core DCE component presented respondents with a series of choice questions. Each choice set consisted of two hypothetical peer support service options, described by varying levels of the eight attributes, alongside an opt-out alternative. Prior to completing the choice tasks, respondents were asked to imagine that they, or someone they cared for with a long-term mental health condition, required peer support and were presented with alternative peer support service packages. Respondents were asked to select the package they would personally prefer in each choice scenario or choose the opt-out option if neither package was preferred. An example of a DCE choice set is shown in figure 1, while the full background information and respondent instructions are provided in the online supplemental material. An optional free response text box was made available at the end of the DCE for feedback.

**Figure 1:**
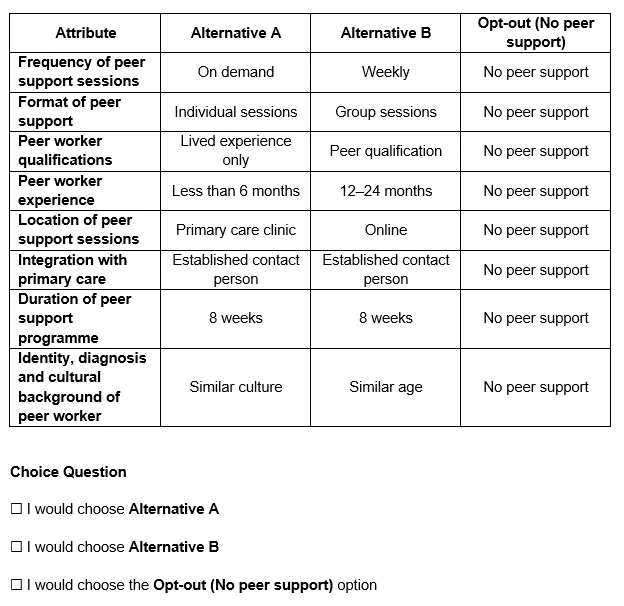
Example choice set.

The survey was developed by a core team of researchers (n=6), some of whom have lived experience with the mental health system as PSRs and/or carers. The survey was discussed in meetings with stakeholders intended to provide guidance and oversight across the project. The DCE was therefore piloted with a range of stakeholders including researchers in relevant fields (n=2), peer workers (n=3), PSRs (n=2), directors of community managed organisations (n=2) and GPs (n=3) to test its clarity, relevance and layout. Pilot testing involved completion of the draft online survey by stakeholders, followed by structured verbal and written feedback on attribute wording, attribute levels, survey instructions, layout and respondent burden. Feedback highlighted the need for improved phrasing of certain questions and the simplification of technical terminology to enhance accessibility. These revisions resulted in the final survey version, which included:

- A preamble defining key terms such as ‘peer support services’ and ‘lived experience’.
- Examples illustrating the attributes and levels to support comprehension.
- Adjusted question layouts to streamline the respondent experience.

The final DCE was designed to capture nuanced preferences for peer support services, allowing for the analysis of trade-offs between attributes and identifying priority areas for service improvement. This process ensured the robustness and relevance of the survey while maintaining a respondent-centred focus.

### DCE design

The experimental design used a fractional factorial approach to minimise cognitive burden while maintaining statistical efficiency.^34^ A D-efficient choice design with zero priors was generated using Ngene software (Ngene V.1.1.1; ChoiceMetrics, 2012) for a multinomial logit model. The resulting design was subsequently analysed using both conditional logit and random-parameter logit (RPL) models, as described in the ‘Statistical and econometric approaches’ section below. To minimise respondent burden, five blocks of eight choice sets were created and respondents were randomly assigned to one block. Each choice set was designed to maximise statistical efficiency while minimising correlations between attribute levels and, where feasible, maintaining level balance. An alternative-specific constant (ASC) captured the baseline utility associated with choosing either peer-support programme rather than opting out. The opt-out alternative, which represented selecting neither hypothetical programme, was used as the reference category. Accordingly, all programme-attribute variables for the opt-out alternative were coded as structural zeros, while the ASC was applied to both peer-support programme alternatives.

### DCE respondents and administration

Expressions of interest to participate in the DCE were invited through the Lived Experience Australia Representative Panel, a national panel of around 200 people (including 70 peer workers) with diverse lived experience backgrounds such as mental health, disability, National Disability Insurance Scheme access, family and domestic violence, alcohol and other drugs, lesbian, gay, bisexual, transgender, intersex, queer, asexual and other sexually or gender diverse (LGBTQIA+) people, culturally and linguistically diverse, First Nations, rural/remote, and youth and older age. At the time, the panel had contributed to over 50 advisory and working groups in the preceding year. This group was chosen to enable maximum variation in lived experience across key variables of age, gender, and location. The call for expressions of interest was left open for 10 days over which time 110 people recorded their names and email addresses. Recruitment remained open until responses plateaued and no further responses were received. Because participation had effectively ceased, reminder invitations were not issued before the survey was closed. The questionnaires were administered via the online portal REDCap in December 2024 over a period of approximately 2 weeks.

Respondents were able to choose one or more descriptions of themselves as a ‘PSR’, ‘carer’ or ‘PSW’. Due to a low number of people describing themselves solely as carers, the PSR and carer categories were collapsed in reference to service receipt and provision.

### Patient and public involvement

The larger project in which this DCE was positioned was lived experience-led with several chief investigators representing the mental health lived experience community, peer service development and delivery, and primary care research and delivery. This group included the project lead who brought two decades of lived experience of mental health peer work and national mental health recipient and carer advocacy. Their involvement in DCE development was complemented by contributions from the project manager who brought their own lived experience PSR expertise and insights from their regular contact with general practice staff, PSWs and peer service managers involved in the larger project. Other members of this core working group included researchers with extensive knowledge and experience of translation of healthcare models to practice in primary and community care.

The DCE attributes were formulated by the core research team, which included lived experience experts in mental health peer work. As described in the ‘Attribute development’ section, this process was informed by three key evidence reviews, including the project lead’s prior comprehensive evidence review of peer workforce models, implementation challenges and enablers for the Australian government Department of Health.^33^ Design of the DCE itself was collaborative, with early draft ideas presented by the core team to the combined Stakeholder and Project Reference Group that informed the larger project’s activities. The reference group included two lived experience experts in the national development and delivery of peer workforce training and workforce guidelines, PSRs with lived experience of mental health challenges, family carers, peer workers, community services stakeholders and the wider research team. DCE co-design with the reference group involved a series of robust discussions and debates about the practical realities and theoretical underpinnings of peer support services. Their comments about the importance of DCE attributes aligning with the core values and principles of peer work were critical in refining the practical foundations of the study and final selection of DCE attributes.^1^

### Statistical and econometric approaches

We generated descriptive statistics for respondents’ characteristics and attitudinal questions. Our econometric approach was based on a standard random utility framework^35^ which assumes that respondents choose the alternative that maximises their utility. The utility (U) of alternative j for individual n in choice set k was specified as:


$$\varepsilon_{njk}$$

where X was a vector of observed attributes, β was the corresponding coefficient (parameter) and $\varepsilon_{njk}$ was an error term which was assumed to be an independent and identically distributed extreme value. In the first instance, a traditional conditional logit (clogit)^36^ was fitted to the data as a benchmark specification under the assumption of homogeneous preferences. We then used the RPL, which accounts for unobserved preference heterogeneity only.^36 37^ An RPL regression model was chosen for this study due to its ability to account for unobserved heterogeneity in preferences among respondents. Unlike simpler models, such as the conditional logit, the RPL accommodates variations in individual choices by allowing coefficients to vary across respondents, thus providing more robust and nuanced insights into preferences.^37^ Several studies have shown that incorrectly restricting preferences to be homogeneous may lead to biased parameter estimates for individuals^38–40^ with policy implications in terms of the optimal implementation of results from a DCE.^39 41^ To limit model complexity relative to the available sample size, a parsimonious RPL specification was developed using a sequential model-building approach. An initial model specified all non-reference attribute-level coefficients as random. Coefficients were subsequently constrained to be fixed where there was limited evidence of unexplained heterogeneity, based on the magnitude and statistical significance of the estimated SD, parameter stability across alternative specifications, model convergence and changes in log likelihood and information criteria. Random coefficients were retained where preference heterogeneity was conceptually plausible and empirically supported. These included selected levels relating to peer-worker qualifications and experience, service location, integration with primary care, programme duration, frequency and mode of delivery.

The final parsimonious RPL included 11 random coefficients, assumed to follow normal distributions, with the remaining attribute coefficients estimated as fixed. The model was estimated by maximum simulated likelihood using 500 scrambled Halton draws. Model performance was assessed using the maximised log likelihood, Akaike information criterion (AIC) and Bayesian information criterion (BIC).^37^ The parsimonious RPL was used as the primary model, with the conditional logit model reported as a benchmark and sensitivity analysis.

Formal comparisons between PSWs and PSRs were undertaken using a pooled unrestricted model in which the respondent group was interacted with the ASC and all attribute coefficients. This approach permits direct statistical testing of subgroup differences while avoiding comparisons of coefficients from separately estimated models, which may be confounded by scale differences. Overall respondent-group differences were assessed using a likelihood-ratio test for the conditional logit models and joint Wald and likelihood-ratio tests for the parsimonious RPL models.

Choice consistency was measured using a test of non-satiation^22^ based on a choice of restaurant (A or B) presented as a practice question. All levels of attributes for restaurant A (in terms of distance from respondent’s home, menu, size of meal and cost) were better than those for restaurant B. Selection of restaurant A was interpreted as passing the dominance test. This test was not based on peer support lived experiences scenarios because the attributes and levels we included within the peer support scenarios did not easily lead to a test of dominance of one scenario over another. All analyses were conducted in Stata/MP V.18 (Stata). A significance level threshold of 5% (0.05) was assumed as the criterion for determining statistical significance in all analyses.^42^ Categorical attributes were dummy coded with reference categories specified for each, allowing the estimated RPL coefficients to be interpreted as relative utility compared with the reference.

### Qualitative analysis of free text responses

A reflexive thematic analysis of the free text responses^43^ was performed from an inductive phenomenological approach by a member of the research team who had not personally received formal peer support but had lived experience of mental health difficulties across different levels of the mental healthcare system. These experiences were considered in the construction of themes as a PSR looking at a prospective form of community-based care while working in a biomedically framed science. Any responses to the question *“*Do you have any comments that you would like to make about this questionnaire?” were analysed, even if the comments related to the format of the study. All responses were intended to be anonymous, however some respondents signed their entries. All respondents were given a code linked to their role as provider or PSR and anonymised for analysis. The approach to qualitative content analysis used in this study has been reported in accordance with the Standards for Reporting Qualitative Research (SRQR).^44^ A transparency and contextual statement is available in the online supplemental material.

## Results

### Respondent characteristics

As detailed in table 1, 84 (76%) of the initial targeted sample of 110 completed the DCE (43 PSWs and 41 PSRs). There were no statistically significant differences in characteristics between DCE completers and non-completers. Most respondents identified as female (73%), and middle-aged respondents (45–64 years) comprised 62% of the sample. Peer workers made up 51% of the sample, and almost all (94%) had prior experience in paid roles, demonstrating their professional experience.

**Table 1:** Respondent characteristics

	Type of respondent		
	Peer support worker	Peer service recipient	Total
N	43 (51%)	41 (49%)	84 (100%)
Gender			
Male	11 (26%)	7 (17%)	18 (21%)
Female	29 (67%)	32 (78%)	61 (73%)
Non-binary	3 (7%)	2 (5%)	5 (6%)
Age, years			
≤19	1 (2%)	0 (0%)	1 (1%)
20–24	1 (2%)	1 (2%)	2 (2%)
25–29	0 (0%)	3 (7%)	3 (4%)
30–34	3 (7%)	0 (0%)	3 (4%)
35–39	3 (7%)	3 (7%)	6 (7%)
40–44	2 (5%)	5 (12%)	7 (8%)
45–49	5 (12%)	4 (10%)	9 (11%)
50–54	14 (33%)	5 (12%)	19 (23%)
55–59	5 (12%)	6 (15%)	11 (13%)
60–64	7 (16%)	6 (15%)	13 (15%)
65–69	1 (2%)	3 (7%)	4 (5%)
70–74	1 (2%)	4 (10%)	5 (6%)
75–79	0 (0%)	1 (2%)	1 (1%)
State			
New South Wales	7 (16%)	11 (27%)	18 (21%)
Australian Capital Territory	1 (2%)	0 (0%)	1 (1%)
Victoria	12 (28%)	9 (22%)	21 (25%)
Tasmania	2 (5%)	2 (5%)	4 (5%)
South Australia	5 (12%)	7 (17%)	12 (14%)
Western Australia	4 (9%)	6 (15%)	10 (12%)
Queensland	12 (28%)	6 (15%)	18 (21%)
Lived experience			
Lived experience of mental health condition	12 (28%)	24 (59%)	36 (43%)
Care for someone living with mental health condition	2 (5%)	12 (29%)	14 (17%)
Peer worker with lived experience of a mental health condition	29 (67%)	5 (12%)	34 (40%)
Worked in paid-lived-experience role?			
No	2 (7%)	0 (0%)	2 (6%)
Yes	27 (93%)	5 (100%)	32 (94%)

### Attitudinal responses

The attitudinal questions demonstrated widespread support for peer support services across all respondent groups (table 2). Among the total sample, 100% agreed that peer support services should be an integral part of mental healthcare and 99% agreed that peer support can complement traditional mental health services. All respondents believed that peer support services positively impact mental health outcomes, and 100% agreed that peer support can reduce the feeling of isolation and loneliness associated with mental health. The ability for PSWs to offer a range of services was also highly valued (94%).

**Table 2:** Attitudinal questions depicting respondents’ thoughts on mental health peer workers being part of primary care

	Type of respondent		
	**Peer** **support worker**	**Peer service** **recipient**	**Total**
N	43 (51%)	41 (49%)	84 (100%)
Peer support services would be more effective if provided in settings or locations outside of GP clinics			
Strongly disagree	0 (0%)	1 (2%)	1 (1%)
Disagree	5 (12%)	7 (17%)	12 (14%)
Neither agree nor disagree	11 (26%)	13 (32%)	24 (29%)
Agree	16 (37%)	13 (32%)	29 (35%)
Strongly agree	11 (26%)	7 (17%)	18 (21%)
The first meeting between patients and peer workers should be face-to-face			
Disagree	2 (5%)	4 (10%)	6 (7%)
Neither agree nor disagree	12 (28%)	8 (20%)	20 (24%)
Agree	12 (28%)	10 (24%)	22 (26%)
Strongly agree	17 (40%)	19 (46%)	36 (43%)
Peer workers should accompany patients when they go to a GP clinic			
Strongly disagree	2 (5%)	0 (0%)	2 (2%)
Disagree	4 (9%)	6 (15%)	10 (12%)
Neither agree nor disagree	19 (44%)	17 (41%)	36 (43%)
Agree	14 (33%)	14 (34%)	28 (33%)
Strongly agree	4 (9%)	4 (10%)	8 (10%)
Peer workers should be able to offer a range of services (eg, emotional support, coping strategies and self-care techniques, goal setting and motivation, navigating systems and services)			
Disagree	0 (0%)	1 (2%)	1 (1%)
Neither agree nor disagree	1 (2%)	3 (7%)	4 (5%)
Agree	7 (16%)	10 (24%)	17 (20%)
Strongly agree	35 (81%)	27 (66%)	62 (74%)
Peer support services can positively impact your mental health			
Agree	5 (12%)	7 (17%)	12 (14%)
Strongly agree	38 (88%)	34 (83%)	72 (86%)
Peer support services can complement traditional mental health services (eg, therapy, counselling)			
Neither agree nor disagree	0 (0%)	1 (2%)	1 (1%)
Agree	7 (16%)	8 (20%)	15 (18%)
Strongly agree	36 (84%)	32 (78%)	68 (81%)
Peer support services should be an integral part of the mental healthcare system			
Agree	4 (9%)	5 (12%)	9 (11%)
Strongly agree	39 (91%)	36 (88%)	75 (89%)
Peers need to have successfully navigated their own mental health challenges to effectively support others			
Disagree	1 (2%)	1 (2%)	2 (2%)
Neither agree nor disagree	5 (12%)	5 (12%)	10 (12%)
Agree	16 (37%)	17 (41%)	33 (39%)
Strongly agree	21 (49%)	18 (44%)	39 (46%)
Peer support services have the power to reduce feelings of isolation and loneliness associated with mental health issues			
Agree	9 (21%)	11 (27%)	20 (24%)
Strongly agree	34 (79%)	30 (73%)	64 (76%)
Peer support services can be as effective as traditional mental health services (eg, therapy, counselling)			
Disagree	0 (0%)	2 (5%)	2 (2%)
Neither agree nor disagree	3 (7%)	4 (10%)	7 (8%)
Agree	15 (35%)	15 (37%)	30 (36%)
Strongly agree	25 (58%)	20 (49%)	45 (54%)
Peer support services can be more effective than traditional mental health services (eg, therapy, counselling)			
Strongly disagree	0 (0%)	1 (2%)	1 (1%)
Disagree	0 (0%)	3 (7%)	3 (4%)
Neither agree nor disagree	14 (33%)	11 (27%)	25 (30%)
Agree	13 (30%)	13 (32%)	26 (31%)
Strongly agree	16 (37%)	13 (32%)	29 (35%)

GP, general practitioner.

Descriptive differences were observed in attitudinal responses relating to delivery settings and engagement strategies. A greater proportion of PSWs agreed that the service would be more effective if provided outside of GP clinics versus PSRs (63% vs 49%). Extending on this, PSRs placed particular emphasis on personal connection, with a higher proportion preferring face-to-face initial meetings than PSWs (71% vs 67%). This was supported by strong endorsement of relational aspects, including peer workers offering a broad range of supports (94%), having lived experience (85%), and reducing isolation and loneliness (100%).

Respondents showed moderate support for the idea that peer workers should accompany patients when attending GP clinics, with 44% of PSRs and 42% of peer workers agreeing or strongly agreeing with this statement. Furthermore, all respondents agreed that peer support services positively impact mental health, and 99% agreed that they can complement traditional mental health services. The belief that peer workers need to have successfully navigated their own mental health challenges was also prevalent, with 49% of PSWs and 44% of PSRs strongly agreeing. Similarly, all respondents agreed that peer support services have the power to reduce feelings of isolation and loneliness.

### Attributes important in choice of peer support packages

The conditional logit model produced qualitatively similar preference estimates but demonstrated poorer overall fit than the revised parsimonious RPL according to the maximised log-likelihood and AIC. Although substantially less complex than the original fully parameterised specification, the parsimonious RPL achieved a higher maximised log likelihood and a lower AIC than the conditional logit model, whereas the BIC favoured the conditional logit because of its stronger penalty for additional parameters (online supplemental table S2). The parsimonious RPL was therefore selected as the primary analytical model, with the conditional logit reported as a benchmark and sensitivity analysis.

Formal subgroup comparisons were conducted using pooled interaction models. Neither the conditional logit nor the parsimonious RPL model showed significant overall preference differences between PSWs and PSRs (conditional logit: LR χ²(24)=34.10, p=0.083; parsimonious RPL: Wald χ²(24)=28.22, p=0.251; LR χ²(24)=32.79, p=0.109; online supplemental table S4). The full-sample parsimonious RPL was therefore retained as the primary analysis (table 3).

**Table 3:** Preferences for characteristics of peer-support programmes: parsimonious RPL model (primary analysis)

Attribute level	Coefficient (SE)
ASC	
Opt-in alternative ASC	−1.604** (0.599)
Peer support worker qualifications	
Peer qualification	0.838** (0.295)
Obtaining qualification	0.350 (0.264)
Lived experience only	Reference
Peer support worker experience of providing	
6–12 months	0.761* (0.328)
12–24 months	0.554 (0.317)
More than 24 months	1.293** (0.407)
Less than 6 months	Reference
Location of peer support sessions	
Primary care clinic	0.601 (0.326)
Community	1.326*** (0.351)
Respondent’s home	0.812* (0.340)
Online	Reference
Peer support integration with primary care	
External referral	0.250 (0.294)
Established contact person*	0.280 (0.352)
Combined in-clinic and external	0.550 (0.288)
Within primary care clinic	Reference
Duration of peer support programme	
8 weeks	0.503 (0.332)
12 weeks	0.866* (0.358)
Ongoing/open-ended	1.281** (0.462)
4 weeks	Reference
Identity, diagnosis and cultural background of peer support provider	
Similar age	0.008 (0.315)
Similar gender	0.005 (0.299)
Similar cultural background	0.094 (0.244)
Similar diagnosis	Reference
Frequency of peer support sessions	
Weekly	1.359** (0.461)
Biweekly	0.737** (0.279)
On-demand	0.816* (0.369)
Monthly	Reference
Format of peer support	
Individual sessions	1.037*** (0.273)
Online chat/forum	0.115 (0.490)
Phone/video calls	1.541* (0.677)
Group sessions	Reference
SD of random parameters	
Peer worker qualifications: peer qualification	0.554 (0.449)
Peer worker experience of providing peer support: more than 24 months	1.014* (0.424)
Location of peer support sessions: community	0.778 (0.458)
Location of peer support sessions: respondent’s home	1.140** (0.373)
Peer support integration with primary care: established contact person	1.232*** (0.345)
Peer support integration with primary care: combined in-clinic and external services	0.463 (0.578)
Duration of peer support programme: ongoing/open-ended	1.244* (0.556)
Frequency of peer support sessions: weekly	0.423 (0.673)
Frequency of peer support sessions: on-demand	1.064** (0.403)
Format of peer support: individual sessions	1.002*** (0.266)
Format of peer support: online chat/forum	1.191 (1.125)
Model statistics	
Observations	2016
Respondents	84
Log likelihood	−515.013
AIC	1100.025
BIC	1296.336

Coefficients are reported with SEs in parentheses. Reference levels are shown as the final row within each attribute. The RPL model included 11 normally distributed random coefficients and was estimated by maximum simulated likelihood using 500 scrambled Halton draws. Estimated SDs of the random parameters are reported as non-negative values. Statistically significant SDs indicate evidence of unobserved preference heterogeneity around the corresponding mean coefficients.

* p<0.05; **p<0.01; ***p<0.001.

* An ‘established contact person’ referred to designated contacts at both the primary care clinic and the peer-support organisation who facilitated communication and continuity between services.

AIC, Akaike information criterion; ASC, alternative-specific constant; BIC, Bayesian information criterion; RPL, random-parameter logit.

The negative opt-in ASC indicated that a hypothetical peer-support programme composed entirely of the reference attribute levels had lower baseline utility than the opt-out alternative, after accounting for the included programme characteristics. This should not be interpreted as an overall preference for opting out: the opt-out alternative was selected in 50 of 672 choice tasks (7.4%), and 17 of 84 respondents selected it at least once (online supplemental table S4). The utility of a peer-support programme also reflects the part-worth utilities associated with its attribute levels, such that positively valued characteristics, such as community-based delivery, more frequent sessions, individual delivery and greater peer-worker experience, can offset the negative baseline ASC and increase programme utility relative to opting out.

Respondents preferred programmes delivered in the community or at the respondent’s home over online delivery. Weekly, biweekly and on-demand peer-support sessions were preferred over monthly sessions, and individual sessions were preferred over group sessions, with phone or video calls also positively valued. Respondents preferred peer workers with formal peer qualifications and greater experience, particularly those with more than 24 months of experience providing peer support. Longer programmes were generally preferred, with 12 weeks and ongoing or open-ended programmes receiving higher utility than 4-week programmes. There was comparatively little evidence that matching PSWs and PSRs by age, gender or cultural background influenced preferences.

Evidence of unobserved preference heterogeneity was observed for several programme characteristics. Significant random-parameter SDs were identified for more than 24 months of peer-support experience, delivery in the respondent’s home, integration through an established contact person, ongoing or open-ended programme duration, on-demand frequency and individual sessions. These findings indicate meaningful variation across respondents in preferences for these programme characteristics and support the use of the parsimonious RPL model.

### Qualitative analysis of free responses

Nearly half (n=41, 48.8%) of the respondents opted to leave comments, with approximately equal representation between PSRs (n=20, 48.8%) and providers (n=21, 51.2%). Four themes were identified from these comments: (1) experiencing frustration with the DCE format, (2) navigating tensions between the medical model and peer work, (3) identifying with multiple roles and (4) contextualising preferences based on individual needs.

#### Experiencing frustration with the DCE format

The primary topic (n=19) of the comments provided was the format of the DCE and survey. While five respondents noted that the survey had been interesting and positive, most who commented on the DCE format (n=14, 73.7%) described their frustration with the options presented. For example, one PSR wrote that it was “VERY [sic] confusing with the packages and options. I ended up opting out of most of them as it was frustrating working out my preferences” (P20: PSR). The key message arising from these comments was that, by its very nature, peer work emphasises flexibility and personalisation, making it uncomfortable and at times incompatible to force respondents to choose between options.

I am a service user, carer and Founding Director of a NFP Peer Service. I couldn’t communicate that in the survey. I also opted out of the scenarios [sic] purely because authentic peer partnerships are about being flexible and personalised in accordance with the participants in the partnership. It could look like any of your options depending [on the partnership]*.* (P16: PSW).

#### Navigating tensions between the medical model and peer work

The second theme highlights the perceived incompatibility between the forced choice format and the ethos of peer work, particularly within primary care settings where mental health is often medicalised. As one PSW noted, “the peer work methodology is the opposite of the medical model, so I don’t think a GP clinic is the best place for it” (P31: Peer worker). Over the years, there has been a strong emphasis on moving away from a strictly biomedical model, with peer workers instead focusing on building person-to-person connections that move beyond diagnostic labels:

Connecting with people on a person to person level regardless of whether [their] label … match[es] the person’s label or not. Peer support needs to challenge a biomedical model of mental health … and not to to [sic] integrate their practice into a medical model, causing moral injury, peer drift and often burnout. Peer work exists to uphold people’s human rights, when a biomedical model often perpetuates human rights violations or doesn’t address them. (R42: PSR).

One PSW also reflected on the tension from a cultural point of view:

As an Aboriginal and Torres Strait Islander man, I found some of the questions a little challenging in terms of supporting first nations people in the space of social and emotional wellbeing. The translation of the biomedical approach walking with the participant to come to self-realisation [sic] [and] making the right choice for the individual and their own recovery journey, we know that supporting first nations people is holistic support. Helping them navigate this space is most important (P04: Peer Worker).

#### Identifying with multiple roles

Respondents expressed a discomfort with being required to align with a single description of experience when many identified with two or more roles. While this forced choice was necessary for the analysis, it became clear that lived experience reflects the wholeness of a person’s life, with individuals drawing on different aspects of their experience interchangeably rather than viewing them as discrete roles.

It’s shame you didn’t have options on the first page to tick all boxes that relate to the person undertaking the survey, as I have all three. Of PLE/Carer/PSW nor that option in any of the programme choices. Some neither agree or disagree is all dependant of a person-centred approach needs basis… respectfully, I wouldn’t choose any of the 8 programmes [in my personal opinion] ‘fit for purpose’ (P20: Peer Worker).

#### Contextualising preferences based on individual needs

Respondents emphasised the importance of services being *“*geared to a specific individual’s needs” (R17: PSR). Respondents differed on what elements were important and discussed how the preferences were not distinct but dependent on broader circumstances as “it really is ideally a combination of elements from each package” (PSR: R49). Several respondents felt that the choice of categories provided to them in the questionnaire were not as important as the *“*quality over quantity” (R39: PSR), in care, choice or connection:

The lived experience based on common domains of culture, age and diagnosis are contentious areas as it also limits a level of connection amongst individuals. Those domains are helpful but dont [sic] necessarily need to be the point of contention (P22: Peer worker).

Others noted that they would not have known what their preferences were before taking part.

Respondents commented on the relative importance of the given categories, such as how being in a rural or metropolitan area changed the context of questions due to the practical local availability of face-to-face services. Features of culture in terms of age and background were also discussed in terms of relative importance, with some advocating for similarities (eg, age related experiences) while others desired the opposite. Similarly, group size, length of support period and a need for qualifications were subjective to personal preference.

## Discussion

This study provides evidence for the acceptability of PSW in mental health and the preferences around its delivery. Positive attitudinal responses across respondent groups showed that peer support is valued to address mental health challenges, and that its inclusion as an additional form of support alongside traditional interventions would be well-received. Although integration coefficients were positive, neither an established contact person nor combined in-clinic and external delivery was statistically significant in the primary parsimonious RPL model. Qualitative comments nevertheless highlighted the perceived importance of communication, continuity and coordinated referral pathways. This finding complements existing literature that highlights ongoing lived experience resistance to biomedical models, as peer services continue to be used to link individuals to medical systems and compliance rather than to support recovery.^5^ In practice, peers working in other levels of care have historically also found it necessary to fight for their values and practices when being integrated into medical systems.^45^ In primary care, the relatively lower preference for clinical settings is notable, particularly given that GPs are often the first point of contact for people seeking mental health support and that mental health clinics are increasingly being integrated into community settings.^12 46^ Respondents consistently preferred peer support delivered in community settings and, to a lesser extent, in respondents’ homes rather than online delivery. Although some differences in coefficient magnitudes were observed between peer workers and PSRs, pooled interaction analyses did not demonstrate statistically significant overall differences in preferences between respondent groups. These findings suggest broad agreement regarding preferred service settings while also indicating meaningful individual-level variation captured by the RPL model. Opting out was uncommon, occurring in 7.4% of choice tasks. The negative opt-in ASC therefore reflects the baseline reference programme rather than a general preference against peer support, suggesting that acceptability depended on programme configuration. This suggests that respondents were broadly supportive of peer support but placed considerable importance on the specific way services were designed and delivered. Some combinations of programme characteristics may therefore have been less attractive than selecting neither programme, even though opting out was uncommon overall.

Differences in preferences for location and modes of delivery highlight the importance of personalising services to accommodate individual needs. Respondents generally preferred community-based and home-based delivery over online delivery, although qualitative comments suggested that online support may be acceptable when face-to-face services are inaccessible. This is consistent with evidence from telehealth studies showing that accessibility and convenience are important determinants of service use.^20 23^ The significant preference heterogeneity observed for several delivery characteristics also suggests that preferences varied considerably between individuals, reinforcing the need for flexible service models rather than a one-size-fits-all approach. Similarly, respondents preferred longer programme durations overall, while the qualitative findings highlighted the importance of balancing sustained therapeutic relationships with flexibility to meet individual circumstances. Together, these findings suggest that peer-support programmes should offer adaptable models of delivery that accommodate the diverse needs and preferences of PSRs.^20^

The preference for PSWs to have, or be in the process of obtaining, a qualification for peer support reflects previous research showing that training supports effective peer work and strengthens professional practice.^16 17^ At the same time, respondents highlighted in their comments that lived experience is the defining feature of peer support and should remain the priority. Taken together, these findings suggest that while qualifications are valued, they are seen as complementing, rather than replacing, lived experience. Respondents also described experienced PSWs as more credible, empathetic and capable of navigating complex mental health systems. These themes closely aligned with the quantitative finding that PSWs with greater experience were consistently preferred across both the conditional logit and parsimonious RPL models.

Similarly, respondents emphasised continuity of care, collaboration with clinicians and coordinated referral pathways as important components of effective peer support. These qualitative findings are directionally consistent with the positive preferences observed for integrated models of peer support, particularly those involving established contact persons or combined in-clinic and external service delivery.

Although some qualitative comments suggested that PSWs and PSRs may place different emphasis on particular aspects of peer support, the pooled interaction analyses did not demonstrate statistically significant overall differences in preferences between respondent groups. The qualitative findings therefore provide explanatory context for the overall preference patterns identified in the DCE rather than evidence of statistically significant subgroup differences. This complements research highlighting the usefulness of learning opportunities about appropriate use of lived experience, reflection on personal limitations and consideration of ability to access support.^7 47^ Ways to achieve this knowledge include—but are not limited to—formal qualifications, which may explain this preference and the apparent diversity seen in respondent responses.

Although formal comparisons did not identify significant overall preference differences between PSWs and PSRs, the parsimonious RPL model demonstrated substantial unexplained preference heterogeneity across individuals. This suggests that variation in preferences is driven more by differences within stakeholder groups than between them. Consequently, flexible peer-support programmes may be more appropriate than models tailored solely according to stakeholder type.

### Implications for policy and practice

The results emphasise the need for tailored service designs that reflect the diverse preferences of PSRs. Provision of services which align with or accommodate mental health PSR preferences has been shown to improve intervention engagement and reduce dropout rates.^48^ In practice, this will mean that PSWs will be diverse in how they offer and provide services, meaning a ‘one-size-fits-all’ approach to funding and care will not be sufficient. Policymakers also need to recognise that peer work involves a cultural shift in mental healthcare, moving away from a strictly medical model towards one that values lived experience and mutual support. As such, clinical settings will need to take care when employing peer workers in order to preserve the PSW ethos. Support is needed to enable the key preferences for implementing peer work services indicated in this study. These include investment in PSW training and formal qualifications, recognition of the value of experienced PSWs, community-based models that address barriers to access and flexible delivery options that accommodate variation in preferred session frequency, duration and format.

### Limitations and future research

While this study provides valuable insights, several limitations warrant consideration. These findings should be considered in the context of the respondents recruited. Recruitment was targeted to ensure that individuals represented the necessary groups and that the sample size could meet the statistical needs of this experiment. It also purposefully valued the professional and experiential expertise of PSWs and PSRs. However, as the high agreement in the attitudinal questions demonstrates, this sample may be especially favourable towards peer work compared with the general population. No reminder invitations were issued during recruitment; additional reminders or a longer recruitment period may have increased participation. These factors, together with the relatively small sample size limit the generalisability of findings. Nevertheless, the consistency of findings between the conditional logit and parsimonious RPL models, together with the absence of significant respondent-group interactions, provides reassurance regarding the robustness of the principal conclusions. The need for assigning categories to each respondent may also have contributed to the variance demonstrated in the study. Respondents referenced the multidimensionality of their perspectives on lived experience, meaning that the division in roles was not necessarily clearly cut and some may have drawn from both perspectives during the study. Future research should explore the long-term outcomes of peer support services and investigate preferences across the broader population of PSRs and peer workers, including in different cultural and socioeconomic contexts.

## Supplementary material

10.1136/bmjopen-2025-112132 online supplemental file 1

10.1136/bmjopen-2025-112132 online supplemental file 2

## Data Availability

Data are available on reasonable request.
