# Supplementary material for: Lived experience preferences for mental health peer support services in Australian primary care: a discrete choice experiment with qualitative analysis
Source: BMJ Open. 2026 Nov 9;16(11):e112132. doi: 10.1136/bmjopen-2025-112132 (PMC13756459; doi:10.1136/bmjopen-2025-112132)
Supplement: Supplementary file 1 [file bmjopen-2025-112132.reviewer_comments.pdf]

PEER REVIEW HISTORY

BMJ Open publishes all reviews undertaken for accepted manuscripts. Reviewers are asked to complete a checklist review form and are provided with free text boxes to elaborate on their assessment. These free text comments are reproduced below.

ARTICLE DETAILS

Title (Provisional)

Lived experience preferences for mental health peer support services in Australian primary care: A discrete choice experiment with thematic analysis

Authors

Kaambwa, Billingsley; Fuss, Belinda; Woods, Taylor-Jade; Rattray, Megan; Shelby-James, Tania; Kaine, Christine; Lawn, Sharon

---

VERSION 1 - REVIEW

**Reviewer**	**1**
**Name**	**Hews-Girard, Julia**
**Affiliation**	**University of Calgary, Faculty of Nursing**
**Date**	**27-Nov-2025**
**COI**	**None**

Thank you for the opportunity to review this manuscript. This continues to be an interesting and timely topic and the paper is, for the most part, clearly written and easy tot follow.

1) In the discussion, pg 20 line 434 - the authors state that some 'participants' expressed concern re: over medicalization. The only quote in the results for this was from a peer support worker. If some of the recipients also provided this same response, I would consider including a quote from a recipient point of view as well. Alternatively, specifying in the discussion which participants tended to express this concern would be helpful to contextualize these results as well as the results showing that peer support workers tended to prefer in-clinic work..

2)on page 21, line 466 and 467 would benefit from some further discussion or rewording for clarity. "This divergence underscores the unique value of continuity in peer support services, where sustained relationships and shared experiences are central to their effectiveness". Continuity is usually associate with long term care relationships - but recipients in this study preferred shorter term relationships. Perhaps clarifying who it is that values continuity? or how continuity is defined would be helpful as I'm not sure that this finding supports the value of continuity.

3) similarly to the previous comments - in talking about certification - which participants value it? are there quotes from both participant groups that support this? Clarifying this would strengthen the conclusions of this work.

4) outside of the discussion, clarifying the outcomes could be helpful as well - e.g., an established contact person.

**Reviewer**	**2**
**Name**	**Loría-Rebolledo, Luis Enrique**
**Affiliation**	**University of Aberdeen**
**Date**	**10-Mar-2026**
**COI**	**None**

This paper describes a DCE study of preferences for peer support in Australia. The study follows state-of-practice in its design and analysis frameworks. Some of the conclusions, specifically those related to differences in preferences across the two groups of interest, are not formally supported by their analysis plan. Appropriate methods to conduct subgroup analysis, using unrestricted models or MRS (although this may not be possible given the model specification) with corresponding statistical tests, are needed to support their conclusions. There are omissions and lack of detail that is needed for the reader to assess the validity of their results. The qualitative component is a nice, albeit potentially underutilised, addition. I provide comments and suggestions below:

The authors use the term consumer, which I understand is commonly used in Australia to refer to patients or service users. However, given the journal's international audience, I recommend that they also reference more widely used international terms.

Given readership of journal, introduction would benefit from formal definitions of the different terms: formal (emphasis on formal vs informal) peer worker, peer consumer, peer support services.

The selection of attributes (and levels) requires more detail. What is a comprehensive literature review? Which papers were identified and do these relate to their empirical question (context, population of interest, research question)? What were the initial sixteen attributes?

Choosing the attributes and levels is key to any DCE. Simply stating that the initial list was reduced "based on relevance, feasibility, and comprehensibility" does not give readers enough information. They need to know how you ensured that no important attributes were left out and that potential interactions were considered. I appreciate there are word limits, but this stage is critical. More transparency and detail should be provided either in the main text or in the supplementary material.

Describe the scenario presented to respondents: this is also part of the DCE choice task.

Describe the pilot stage: were these one-to-one interviews (i.e., think alouds) or sending the survey out and getting follow-up feedback? Incidentally, the detailed changes resulting from the pilot could be put elsewhere, with that word count used to better describe the attribute and task derivation.

Experimental design (Line 200): it is not Dz-error (unless achieving efficiency for a subset of parameters with vector z, which I presume was not the case), its D-error. Describe the target analysis model for which they sought efficiency (I assume its MNL).

In an efficiency design, by definition, orthogonality is not going to be achieved, so the statement in Line 203 is incorrect. It should read ensure minimal correlations (within or across alternatives, I presume it was the former).

Line 206, should also include (and correct as necessary), "…including an alternative specific constant capturing the likelihood of choosing the opt out alternative".

Re the respondent recruitment, is there a specific reason why the recruitment period was so short and no reminders were sent?

Line 223-226 should go in the Introduction and see my earlier comment re definitions.

Line 265, please clarify the mixed logit model was a random-parameter logit model or assuming random distributions on parameters.

The authors correctly note that in *some cases* a mixed logit model (more precisely, a random-parameter logit, RPL) can relax the assumption of preference homogeneity and potentially lead to less biased parameter estimates. Whether the model captures taste or scale heterogeneity is a separate issue that is not explored in this study. However, the choice of model specification and its empirical performance are also constrained by the available sample size and its composition. In this case, the authors estimate an RPL model with 24 parameters using 500 Halton draws on a relatively small sample (approx. 40-ish with 8 sets each), which raises concerns about the feasibility and reliability of such a complex specification. It would not be surprising if the main results were robust to simpler modelling approaches, such as an error-components or even the conditional logit model. Moreover, given that the research question focuses primarily on observed variables, modelling heterogeneity through observed covariates within a simpler framework may provide a more parsimonious and transparent way to generate the empirical evidence. Can the authors report the results from a conditional logit model and reflect on whether the use of an RPL specification is necessary or appropriate for addressing their research question.

The ASC (related to choosing opt-out alternative)'s magnitude stands out as it contrasts with what seems to be revealed preference. Related to this, the statement in Line 333 "participants showed a positive preference for the opt-out option" is not technically correct.

Given the confounding of the base levels of the dummy coded attributes, I suspect the proposed specification is not reflecting the author's conclusions: can the authors confirm the robustness of their results when specifying the ASC as the opt-in alternatives, as this provides a more intuitive interpretation. Can the authors also report the choice proportions (how many tasks were opted out and how many times respondents opted out), as this may provide some clarity for this estimate.

The authors are doing subgroup preference comparisons by what seems to be a comparison of the parameter estimate magnitudes: this is incorrect as differences may be due to scale. The formal way to compare preferences is through marginal rates of substitution or via interactions in an unrestricted specification. Can the authors also confirm any unrestricted model leads to better model fit using formal likelihood ratio tests.

The qualitative component is a nice, and seldomly explored in DCEs, contribution, but it feels disjointed from the preference results. I suggest the authors try to reflect on their preferences and try to pick out if there's any internal validity of the subgroup analysis results (which seems to be one of their main research questions) based on the qualitative data obtained. For example, are peer workers signalling a higher sensitivity to experience and integration. I appreciate there may be limited data, but this would significantly enhance the validity of the results and provide insights that can truly contribute to the literature.

The presentation of the task with the opt-out alternative levels each defined as "No Preference" is unusual and may inadvertently be interpreted as indifference rather than zero part-worth utility, which could be problematic from a modelling perspective. I assume this was pretested in the pilot to ensure the opt-out was interpreted as intended. But I raise this given the ASC's comment above. Can the authors reflect on this and incorporate to manuscript as appropriate.

VERSION 1 - AUTHOR RESPONSE

Reviewer: 1

Dr. Julia Hews-Girard, University of Calgary

Comments to the Author:

**Comments to the Author**	**Authors' responses to comments**
Thank you for the opportunity to review this manuscript. This continues to be an interesting and timely topic and the paper is, for the most part, clearly written and easy tot follow.	We thank the author for their positive feedback.
1) In the discussion, pg 20 line 434 - the authors state that some 'participants' expressed concern re: over medicalization. The	Thank you for this comment. We have extended the next quote under the line you are referring to, which also

only quote in the results for this was from a peer support worker. If some of the recipients also provided this same response, I would consider including a quote from a recipient point of view as well. Alternatively, specifying in the discussion which participants tended to express this concern would be helpful to contextualize these results as well as the results showing that peer support workers tended to prefer in-clinic work..	discussed the need to move away from the medicalisation of mental health from a recipient perspective.<br><br>**Added text (Results, lines 485 – 490)**<br>"Connecting with people on a person to person level regardless of whether [their] label … match[es] the person's label or not. Peer support needs to challenge a biomedical model of mental health … and not to to [sic] integrate their practice into a medical model, causing moral injury, peer drift and often burnout. Peer work exists to uphold people's human rights, when a biomedical model often perpetuates human rights violations or doesn't address them." (R42: PSR).
2)on page 21, line 466 and 467 would benefit from some further discussion or rewording for clarity. "This divergence underscores the unique value of continuity in peer support services, where sustained relationships and shared experiences are central to their effectiveness". Continuity is usually associate with long term care relationships - but recipients in this study preferred shorter term relationships. Perhaps clarifying who it is that values continuity? or how continuity is defined would be helpful as I'm not sure that this finding supports the value of continuity.	Thank you for highlighting this issue. Following the reviewer's and Reviewer 2's comments, we replaced the separate subgroup comparisons with formal pooled interaction analyses. These analyses did not identify statistically significant overall preference differences between peer workers and recipients.<br><br>We have therefore removed the statement suggesting that recipients preferred shorter relationships while peer workers preferred sustained relationships. The revised Discussion now focuses on the full-sample finding that 12-week and ongoing/open-ended programmes were preferred to four-week programmes.

3) similarly to the previous comments - in talking about certification - which participants value it? are there quotes from both participant groups that support this? Clarifying this would strengthen the conclusions of this work.	We have revised this section in light of the pooled interaction analyses, which did not identify significant overall differences between respondent groups. The Discussion now states that participants preferred formally qualified peer workers, while the qualitative findings indicate that qualifications were viewed as complementing, rather than replacing, lived experience.
4) outside of the discussion, clarifying the outcomes could be helpful as well - e.g., an established contact person.	We have clarified this term as a footnote in Table 3 (page 20).<br><br>" An "established contact person" referred to designated contacts at the primary care clinic and peer-support organisation who were responsible for facilitating communication and continuity between services."

Reviewer: 2

Dr. Luis Enrique Loría-Rebolledo, University of Aberdeen

**Comments to the Author**	**Authors' responses to comments**
This paper describes a DCE study of preferences for peer support in Australia. The study follows state-of-practice in its design and analysis frameworks. Some of the conclusions, specifically those related to differences in preferences across the two groups of interest, are not formally supported by their analysis plan. Appropriate methods	We thank the reviewer for their careful consideration of the manuscript. We respond to each comment in turn below.

to conduct subgroup analysis, using unrestricted models or MRS (although this may not be possible given the model specification) with corresponding statistical tests, are needed to support their conclusions. There are omissions and lack of detail that is needed for the reader to assess the validity of their results. The qualitative component is a nice, albeit potentially underutilised, addition. I provide comments and suggestions below:	
The authors use the term consumer, which I understand is commonly used in Australia to refer to patients or service users. However, given the journal's international audience, I recommend that they also reference more widely used international terms.	Thank you for this helpful suggestion. We agree that the term "consumer," while commonly used in the Australian mental health context, may be less familiar to an international readership. We have therefore revised the manuscript to use the more widely understood term "peer service recipient (PSR)" throughout. The term "consumer" is now retained only at its first mention in the Introduction, where we explain that it is commonly used in Australia as an alternative term for a PSR.<br><br>**Edited text (Introduction, lines 45 - 47)**<br>"Shared lived experiences between a formal 'peer support worker' (PSW) and a peer service recipient (PSR), also referred to as a 'consumer' in the Australian context, form the backbone of peer support services."
Given readership of journal, introduction would benefit from formal definitions of the different terms: formal	Thank you for this helpful suggestion. We agree that clearer definitions would assist the journal's international readership. We have therefore added formal definitions in the Introduction to distinguish formal peer support work from informal peer support

(emphasis on formal vs informal) peer worker, peer consumer, peer support services.	and to clarify the terminology used for those receiving peer support.<br><br>**Added Text (Introduction, lines 45 - 55)**<br>"Shared lived experiences between a formal 'peer support worker' (PSW) and a peer service recipient (PSR), also referred to as a 'consumer' in the Australian context, form the backbone of peer support services.[1] ... In this paper, PSWs refer to individuals employed or engaged in designated lived experience roles who intentionally use their lived experience of mental health challenges, recovery, and/or caring for someone experiencing mental ill-health to support others. This differs from 'informal peer support', which occurs naturally between people with shared experiences outside a designated service role. We use the term 'peer service recipient' to describe individuals receiving support from a formal PSW in a mental health or primary care setting.[1]"<br><br>**Reference**<br>1. Byrne L, Wang L, Roennfeldt H, Chapman M, Darwin L, Castles C, Craze L, Saunders M. National Lived Experience (Peer) Workforce Development Guidelines. Canberra: National Mental Health Commission; 2021. Available from: https://www.mentalhealthcommission.gov.au/publications/national-lived-experience-peer-workforce-development-guidelines (accessed 10 July 2026)
The selection of attributes (and levels) requires more detail. What is a comprehensive literature review? Which papers were identified and do these relate to their empirical question (context, population of interest, research question)? What	Thank you for this helpful suggestion. We agree that the attribute development process required greater transparency and have substantially revised the manuscript to provide additional detail.<br><br>First, we have expanded the 'Attribute Development' section (lines 149 – 182) to clarify what was meant by the "comprehensive literature review." Specifically, we now explain that the evidence synthesis drew primarily on three reviews considered most relevant to the objectives of this DCE:

were the initial sixteen attributes?	• Repper and Carter's narrative review of peer support in mental health services, which describes the defining principles and core components of peer support interventions and peer work.[7]<br>• Lloyd-Evans et al.'s systematic review and meta-analysis of peer support interventions for people with severe mental illness, which synthesises evidence on the characteristics, delivery models, and effectiveness of formal peer support services.[32]<br>• Lawn et al.'s rapid review and environmental scan, commissioned by the Australian Government Department of Health, which examined contemporary peer workforce models, implementation challenges, and innovative service delivery approaches relevant to the Australian primary care context.[33]<br><br>We have clarified that these reviews were selected because they collectively addressed the context, population, and service delivery questions underpinning our DCE, including the defining characteristics of peer support, peer workforce qualifications and experience, models of service delivery, and implementation within mental health, primary care, and community settings. We have also noted that the project lead had previously led the Australian Government rapid review, which provided an important evidence base for identifying candidate attributes.<br><br>Second, we have clarified that the evidence-informed candidate attributes were not selected from the literature alone. Rather, they were iteratively refined through discussions with the multidisciplinary research team and a lived experience-led steering group comprising peer service recipientss with lived experience of mental health challenges, peer workers, family carers, service providers, and primary care researchers. We now describe the criteria used to refine the attributes, including relevance to the study objectives, alignment with the core principles of peer support, policy and practice importance,

	feasibility for inclusion in a DCE, comprehensibility for respondents, potential overlap between attributes, and respondent burden.<br><br>Finally, to improve transparency, we have added Supplementary Table S1, which presents the 16 initial candidate attributes, the primary evidence sources informing each attribute, whether the attribute was retained in the final DCE, how retained attributes were refined, and the rationale for exclusion where applicable.<br><br>We believe these revisions provide a much clearer description of the attribute development process and demonstrate that the final DCE attributes were informed by both the best available evidence and a rigorous lived experience-led co-design process.<br><br>**Added Text (Attribute Dvelopment , lines 144 - 171)**<br>"Attributes were developed iteratively through a rigorous process that combined evidence synthesis with stakeholder co-design.<br><br>The evidence synthesis drew primarily on three reviews considered most relevant to the objectives of this DCE: a narrative review of peer support in mental health services, [7] a systematic review and meta-analysis of peer support interventions for people with severe mental illness,32 and a rapid review and environmental scan of peer workforce models, implementation challenges, and innovative service delivery approaches commissioned by the Australian Government Department of Health.[33] Together, these reviews synthesised evidence on the defining characteristics of peer support, PSW qualifications and experience, models of service delivery, and implementation within mental health, primary care, and community settings. The project lead had previously led the Australian Government rapid review of peer workforce models and implementation,[33] and this work provided an important evidence base for identifying candidate attributes relevant to contemporary peer support services.

	Drawing on these evidence sources, an initial list of 16 candidate attributes was identified. The preliminary attribute list was subsequently refined through iterative discussions with the multidisciplinary research team and a steering group comprising PSRs with lived experience of mental health challenges, PSWs, family carers, service providers, and primary care researchers. Candidate attributes were considered according to their relevance to the study objectives, alignment with the core principles of peer support, policy and practice importance, feasibility for inclusion in a DCE, comprehensibility for respondents, potential overlap with other attributes, and respondent burden. Potential conceptual overlap between candidate attributes was also considered during refinement to minimise redundancy while ensuring that the principal dimensions of peer support service delivery were represented. This iterative process resulted in the selection of eight attributes representing meaningful and actionable variations in peer support service delivery. Supplementary Table S1 presents the initial 16 candidate attributes, the primary evidence sources informing each attribute, and the rationale for their retention, modification, or exclusion."<br><br>**References**<br>7. Repper J, Carter T. A review of the literature on peer support in mental health services. J Ment Health. 2011;20(4):392-411.<br>32. Lloyd-Evans B, Mayo-Wilson E, Harrison B, Istead H, Brown E, Pilling S, et al. A systematic review and meta-analysis of randomised controlled trials of peer support for people with severe mental illness. BMC Psychiatry. 2014;14:39.<br>33. Lawn S, Dee-Price B, Mohammadi L, Kaine C. Rapid review of international peer-reviewed literature, and targeted environmental scan of the grey literature, to identify innovative service delivery models to support increased engagement of the peer workforce. Adelaide: Lived Experience Australia Ltd; 2024.

Choosing the attributes and levels is key to any DCE. Simply stating that the initial list was reduced “based on relevance, feasibility, and comprehensibility” does not give readers enough information. They need to know how you ensured that no important attributes were left out and that potential interactions were considered. I appreciate there are word limits, but this stage is critical. More transparency and detail should be provided either in the main text or in the supplementary material.	We agree. As described in our response above, we expanded the Attribute development section and added Supplementary Table S1 documenting all 16 candidate attributes, their evidence sources, refinement and reasons for exclusion. We also clarified that conceptual overlap and potential redundancy were explicitly considered when selecting the final attributes. The following sentence has been added..<br><br>**Added text (Attribute Development, lines 162 - 171):**<br>"Candidate attributes were considered according to their relevance to the study objectives, alignment with the core principles of peer support, policy and practice importance, feasibility for inclusion in a DCE, comprehensibility for respondents, potential overlap with other attributes, and respondent burden. Potential conceptual overlap between candidate attributes was also considered during refinement to minimise redundancy while ensuring that the principal dimensions of peer support service delivery were represented. This iterative process resulted in the selection of eight attributes representing meaningful and actionable variations in peer support service delivery. Supplementary Table S1 presents the initial 16 candidate attributes, the primary evidence sources informing each attribute, and the rationale for their retention, modification, or exclusion."
Describe the scenario presented to respondents: this is also part of the DCE choice task.	Thank you for this helpful suggestion. We agree that the scenario presented to respondents is an important component of the DCE choice task and have expanded the manuscript accordingly.<br><br>In the Methods section, we now clarify that participants were asked to imagine that they, or someone they cared for with a long-term mental health condition, required peer support and were presented with alternative peer support service packages. Participants were asked to select the package they would personally prefer in each choice scenario or choose the opt-out option if neither package was preferred. We have expanded the Methods to describe the scenario.

	**Added text (Methods, lines 203 - 209):**<br>"Prior to completing the choice tasks, respondents were asked to imagine that they, or someone they cared for with a long-term mental health condition, required peer support and were presented with alternative peer support service packages. Respondents were asked to select the package they would personally prefer in each choice scenario or choose the opt-out option if neither package was preferred. An example of a DCE choice set is shown in Figure 1, while the full background information and respondent instructions are provided in the Supplementary Material."<br><br>We have also added the verbatim background information, participant instructions and example choice set to the Supplementary Material.
Describe the pilot stage: were these one-to-one interviews (i.e., think alouds) or sending the survey out and getting follow-up feedback? Incidentally, the detailed changes resulting from the pilot could be put elsewhere, with that word count used to better describe the attribute and task derivation.	We have clarified the pilot procedures. The pilot consisted of stakeholders completing the draft online survey followed by structured verbal and written feedback regarding the clarity of the attributes, attribute levels, instructions and survey layout. Formal think-aloud interviews were not undertaken.<br><br>**Added Text (Survey development and pilot testing, lines 216 - 220):**<br>" Pilot testing involved completion of the draft online survey by stakeholders, followed by structured verbal and written feedback on attribute wording, attribute levels, survey instructions, layout and respondent burden. Feedback highlighted the need for improved phrasing of certain questions and the simplification of technical terminology to enhance accessibility."
Experimental design (Line 200): it is not Dz-error (unless achieving efficiency for a subset of parameters with vector z, which I presume was not the case),	We thank the reviewer for identifying this error. We have corrected the reference from Dz-error to D-error and clarified that the D-efficient design was generated for a multinomial logit (MNL) model using zero priors. The resulting design was subsequently used to estimate both the conditional logit and random-parameter logit (RPL) models.

its D-error. Describe the target analysis model for which they sought efficiency (I assume its MNL).	**Revised text (DCE design, lines 232 - 236)**<br>"A D-efficient choice design with zero priors was generated using Ngene software (Ngene version 1.1.1; ChoiceMetrics Pty Ltd, 2012) for a multinomial logit model. The resulting design was subsequently analysed using both conditional logit and random-parameter logit (RPL) models, as described in the 'Statistical and econometric approaches' section below."
In an efficiency design, by definition, orthogonality is not going to be achieved, so the statement in Line 203 is incorrect. It should read ensure minimal correlations (within or across alternatives, I presume it was the former).	We thank the reviewer for this helpful clarification. We agree that orthogonality is not expected in a D-efficient design and have therefore removed the statement referring to orthogonality. The revised text above now provides a concise description of the D-efficient experimental design without making additional assumptions about its statistical properties.
Line 206, should also include (and correct as necessary), "…including an alternative specific constant capturing the likelihood of choosing the opt out alternative".	We have revised the model specification to explicitly describe the ASC.<br><br>**Added Text (DCE design, lines 239 – 244)**<br>"An alternative-specific constant (ASC) captured the baseline utility associated with choosing either peer-support programme rather than opting out. The opt-out alternative, which represented selecting neither hypothetical programme, was used as the reference category. Accordingly, all programme-attribute variables for the opt-out alternative were coded as structural zeros, while the ASC was applied to both peer-support programme alternatives."
Re the respondent recruitment, is there a specific reason why the recruitment period was so	Recruitment remained open until responses had plateaued and no additional responses were being received. Although the period during which responses were actively received was relatively short, it yielded a substantial proportion of the eligible participants available through the recruitment panel. As no further responses

short and no reminders were sent?	were received after this point, recruitment was considered complete and the survey was closed to enable random allocation of respondents to the choice blocks and commencement of the DCE.<br><br>We acknowledge that reminder invitations may have encouraged additional participation and have noted this as a limitation of the recruitment process in the revised manuscript.<br><br>**Added text (Methods, lines 256 - 258)**<br>"Recruitment remained open until responses plateaued and no further responses were received. Because participation had effectively ceased, reminder invitations were not issued before the survey was closed."<br><br>**Added text (Limitations and future research, lines 629 - 631)**<br>"No reminder invitations were issued during recruitment; additional reminders or a longer recruitment period may have increased participation."
Line 223-226 should go in the Introduction and see my earlier comment re definitions.	We agree and have moved this explanatory material to the Introduction where the terminology is first introduced.
Line 265, please clarify the mixed logit model was a random-parameter logit model or assuming random distributions on parameters.	We have clarified that the mixed logit model was specified as a random-parameter logit model**.**<br><br>**Revised text (DCE design, lines 232 - 236)**<br>" A D-efficient choice design with zero priors was generated using Ngene software (Ngene version 1.1.1; ChoiceMetrics Pty Ltd, 2012) for a multinomial logit model. The resulting design was subsequently analysed using both conditional logit and random-parameter logit (RPL) models, as described in the 'Statistical and econometric approaches' section below."

	**Revised text (Statistical and econometric approaches, lines 306 - 310)**<br>"In the first instance, a traditional conditional logit (clogit)[36] was fitted to the data as a benchmark specification under the assumption of homogeneous preferences. We then used the RPL, which accounts for unobserved preference heterogeneity only.[37] An RPL regression model was chosen for this study due to its ability to account for unobserved heterogeneity in preferences among respondents."
The authors correctly note that in *some cases* a mixed logit model (more precisely, a random-parameter logit, RPL) can relax the assumption of preference homogeneity and potentially lead to less biased parameter estimates. Whether the model captures taste or scale heterogeneity is a separate issue that is not explored in this study. However, the choice of model specification and its empirical performance are also constrained by the available sample size and its composition. In this case, the authors estimate an RPL model with 24 parameters using 500 Halton draws on a relatively small sample (approx. 40-ish with 8 sets each), which raises concerns about the feasibility and reliability of	We thank the reviewer for this thoughtful and constructive comment.<br><br>We would first like to clarify that a conditional logit (CL) model had already been estimated as part of the original analysis. However, because it showed poorer fit than the RPL model according to the maximised log likelihood and AIC, it was not reported in the original submission. Although the BIC favoured the CL model because of its stronger penalty for additional parameters, we recognise that this comparison was not transparent in the manuscript. We have therefore added the CL results in Supplementary Table S2 as a benchmark against which the RPL findings can be evaluated.<br><br>More importantly, we agree with the reviewer's concern that the original fully parameterised RPL specification was likely overly complex relative to the available sample size. Rather than simply reverting to the conditional logit model, we therefore substantially revised the RPL specification itself.<br><br>Specifically, we adopted a parsimonious model-building strategy. We first estimated the original fully parameterised RPL model in which all non-reference attribute levels were specified as random. We then sequentially constrained coefficients exhibiting little evidence of unexplained preference heterogeneity (based primarily on the estimated random-parameter standard deviations, parameter stability across specifications, and their contribution to

<table>
<tr>
<td>such a complex specification. It would not be surprising if the main results were robust to simpler modelling approaches, such as an error-components or even the conditional logit model. Moreover, given that the research question focuses primarily on observed variables, modelling heterogeneity through observed covariates within a simpler framework may provide a more parsimonious and transparent way to generate the empirical evidence. Can the authors report the results from a conditional logit model and reflect on whether the use of an RPL specification is necessary or appropriate for addressing their research question.</td>
<td>overall model fit) while retaining random coefficients for attributes where heterogeneity was both theoretically plausible and empirically supported. Theoretically, we anticipated that preferences would vary most for attributes reflecting personal service preferences (such as programme location, duration, frequency, mode of delivery, peer-worker experience and integration) whereas preferences for other programme characteristics were expected to be more homogeneous across respondents. Following each simplification, the model was re-estimated and evaluated using convergence, parameter stability, log likelihood and information criteria. This iterative process resulted in a substantially more parsimonious specification while retaining the principal sources of preference heterogeneity.

The revised parsimonious RPL model (Table 3) included 11 random coefficients, was estimated by maximum simulated likelihood using 500 scrambled Halton draws, and converged successfully.

To evaluate whether retaining the RPL framework remained justified, we compared the revised parsimonious RPL with the conditional logit model (Supplementary Table S2).
<table>
<tr><th>Model</th><th>Log likelihood</th><th>AIC</th><th>BIC</th></tr>
<tr><td>Conditional logit</td><td>−535.97</td><td>1119.94</td><td>1254.55</td></tr>
<tr><td>Parsimonious RPL</td><td>−515.013</td><td>1100.025</td><td>1296.336</td></tr>
</table>
Although considerably simpler than the original fully parameterised specification, the parsimonious RPL continued to achieve a higher maximised log likelihood and a lower AIC than the conditional logit model, indicating improved model fit after accounting for unobserved preference heterogeneity. As expected, the BIC favoured the conditional logit model because of its stronger penalty for additional parameters.</td>
</tr>
</table>

	Importantly, the substantive conclusions were highly consistent across the conditional logit, the original fully parameterised RPL, and the revised parsimonious RPL models. We have therefore retained the parsimonious RPL as the primary analytical model while now presenting the conditional logit model as a benchmark and secondary robustness analysis. We believe this revised modelling strategy directly addresses the reviewer's concerns by substantially reducing model complexity while demonstrating that accounting for preference heterogeneity continues to improve model performance.<br><br>**Added text (Statistical and econometric approaches, lines 340 - 369)**<br>"In the first instance, a traditional conditional logit (clogit)[36] was fitted to the data as a benchmark specification under the assumption of homogeneous preferences. We then used the RPL, which accounts for unobserved preference heterogeneity only.[37] An RPL regression model was chosen for this study due to its ability to account for unobserved heterogeneity in preferences among respondents. Unlike simpler models, such as the conditional logit, the RPL accommodates variations in individual choices by allowing coefficients to vary across respondents, thus providing more robust and nuanced insights into preferences.[37] Several studies have shown that incorrectly restricting preferences to be homogeneous may lead to biased parameter estimates for individuals [38-40] with policy implications in terms of the optimal implementation of results from a DCE.[39, 41] To limit model complexity relative to the available sample size, a parsimonious RPL specification was developed using a sequential model-building approach. An initial model specified all non-reference attribute-level coefficients as random. Coefficients were subsequently constrained to be fixed where there was limited evidence of unexplained heterogeneity, based on the magnitude and statistical significance of the estimated standard deviations, parameter stability across alternative specifications, model convergence, and changes in log likelihood and information

	criteria. Random coefficients were retained where preference heterogeneity was conceptually plausible and empirically supported. These included selected levels relating to peer-worker qualifications and experience, service location, integration with primary care, programme duration, frequency, and mode of delivery.<br><br>The final parsimonious RPL included 11 random coefficients, assumed to follow normal distributions, with the remaining attribute coefficients estimated as fixed. The model was estimated by maximum simulated likelihood using 500 scrambled Halton draws. Model performance was assessed using the maximised log likelihood, Akaike information criterion and Bayesian information criterion [37]. The parsimonious RPL was used as the primary model, with the conditional logit model reported as a benchmark and sensitivity analysis."<br><br><br>**Added and edited text (Results, lines 408 - 436)**<br>"The conditional logit model produced qualitatively similar preference estimates but demonstrated poorer overall fit than the revised parsimonious RPL according to the maximised log-likelihood and AIC. Although substantially less complex than the original fully parameterised specification, the parsimonious RPL achieved a higher maximised log likelihood and a lower AIC than the conditional logit model, whereas the BIC favoured the conditional logit because of its stronger penalty for additional parameters (Supplementary Table S2). The parsimonious RPL was therefore selected as the primary analytical model, with the conditional logit reported as a benchmark and sensitivity analysis. Formal subgroup comparisons were conducted using pooled interaction models. Neither the conditional logit nor the parsimonious RPL model showed significant overall preference differences between PSWs and PSRs (conditional logit: LR $\chi^2(24)=34.10$, $p=0.083$; parsimonious RPL: Wald $\chi^2(24)=28.22$, $p=0.251$; LR $\chi^2(24)=32.79$, $p=0.109$; Supplementary Table S4).

	The full-sample parsimonious RPL was therefore retained as the primary analysis (Table 3).<br><br>The negative opt-in alternative-specific constant indicated that a hypothetical peer-support programme composed entirely of the reference attribute levels had lower baseline utility than the opt-out alternative, after accounting for the included programme characteristics. This should not be interpreted as an overall preference for opting out: the opt-out alternative was selected in 50 of 672 choice tasks (7.4%), and 17 of 84 respondents selected it at least once (Supplementary Table S3).<br><br>Respondents preferred programmes delivered in the community or at the respondent's home over online delivery. Weekly, bi-weekly and on-demand peer-support sessions were preferred over monthly sessions, and individual sessions were preferred over group sessions, with phone or video calls also positively valued. Respondents preferred peer workers with formal peer qualifications and greater experience, particularly those with more than 24 months of experience providing peer support. Longer programmes were generally preferred, with 12-week and ongoing or open-ended programmes receiving higher utility than four-week programmes. There was comparatively little evidence that matching PSWs and PSRs according to age, gender or cultural background influenced preferences."<br><br>**References**<br>36. Greene W. Econometric Analysis. 7 ed. Upper Saddle River, NJ: Pearson; 2012.<br>37. McFadden D, Train K. Mixed MNL models for discrete response. J Appl Econom. 2000;15(5):447-70.<br>38. Breffle WS, Morey ER. Investigating preference heterogeneity in a repeated discrete-choice recreation demand model of Atlantic salmon fishing. Mar Res Econ. 2000;15(1):1-20.

	39. Fiebig DG, Keane MP, Louviere J, Wasi N. The generalized multinomial logit model: Accounting for scale and coefficient heterogeneity. Mark Sci. 2009;29(3):393-421.<br>40. Flynn TN, Louviere JJ, Peters TJ, Coast J. Using discrete choice experiments to understand preferences for quality of life. Variance-scale heterogeneity matters. Soc Sci Med. 2010;70(12):1957-65.<br>41. Mentzakis E, Ryan M, McNamee P. Using discrete choice experiments to value informal care tasks: Exploring preference heterogeneity. Health Econ. 2011;20(8):930-44.
The ASC (related to choosing opt-out alternative)'s magnitude stands out as it contrasts with what seems to be revealed preference. Related to this, the statement in Line 333 "participants showed a positive preference for the opt-out option" is not technically correct. Given the confounding of the base levels of the dummy coded attributes, I suspect the proposed specification is not reflecting the author's conclusions: can the authors confirm the robustness of their results when specifying the ASC as the opt-in alternatives, as this provides a more intuitive interpretation. Can the authors also report the	We thank the reviewer for highlighting this important issue. We agree that our previous interpretation of the alternative-specific constant (ASC) was imprecise.<br><br>To improve interpretability, all econometric models have been reparameterised so that the ASC now represents the baseline utility associated with choosing either of the two peer-support programmes relative to selecting the opt-out alternative. The opt-out alternative therefore serves as the reference category, while all attribute levels associated with the opt-out alternative are coded as structural zeros because no programme attributes apply when respondents select neither programme.<br><br>Under this specification, a negative opt-in ASC should not be interpreted as evidence that respondents generally preferred opting out. Rather, it reflects the utility associated with a hypothetical programme composed entirely of reference attribute levels relative to selecting neither programme.<br><br>To confirm the robustness of this interpretation, we estimated an additional sensitivity analysis including separate ASCs for Alternatives 1 and 2. These constants were statistically indistinguishable, supporting the use of a single common opt-in ASC (i.e. conditional logit: $\chi^2(1)=0.02$, $p=0.879$; parsimonious RPL: $\chi^2(1)=0.13$, $p=0.716$).

<table>
<tr>
<td>choice proportions (how many tasks were opted out and how many times respondents opted out), as this may provide some clarity for this estimate.</td>
<td>As requested, we have also added descriptive statistics describing observed opt-out behaviour.

Added Table: Supplementary Table S3. Observed opt-out behaviour
<table>
<tr><th>Measure</th><th>Value</th></tr>
<tr><td>Respondents</td><td>84</td></tr>
<tr><td>Choice tasks</td><td>672</td></tr>
<tr><td>Opt-out choices</td><td>50 (7.4%)</td></tr>
<tr><td>Respondents selecting opt-out at least once</td><td>17 (20.2%)</td></tr>
<tr><td>Respondents never selecting opt-out</td><td>67 (79.8%)</td></tr>
</table>

The manuscript has been revised accordingly, and the previous statement that participants "showed a positive preference for the opt-out option" has been removed.

Added text (Methods, lines 239 - 244)
“An alternative-specific constant (ASC) captured the baseline utility associated with choosing either peer-support programme rather than opting out. The opt-out alternative, which represented selecting neither hypothetical programme, was used as the reference category. Accordingly, all programme-attribute variables for the opt-out alternative were coded as structural zeros, while the ASC was applied to both peer-support programme alternatives.”

Added text (Results, lines 422 - 427)
“The negative opt-in alternative-specific constant indicated that a hypothetical peer-support programme composed entirely of the reference attribute levels had lower baseline utility than the opt-out alternative, after accounting for the included programme characteristics. This should not be interpreted as an overall preference for opting out: the opt-out alternative was selected in 50 of 672 choice tasks (7.4%), and 17 of 84 respondents selected it at least once (Supplementary Table S3).”</td>
</tr>
</table>

The authors are doing subgroup preference comparisons by what seems to be a comparison of the parameter estimate magnitudes: this is incorrect as differences may be due to scale. The formal way to compare preferences is through marginal rates of substitution or via interactions in an unrestricted specification. Can the authors also confirm any unrestricted model leads to better model fit using formal likelihood ratio tests.	We thank the reviewer for this important methodological observation and agree that direct comparison of coefficients from separately estimated subgroup models may be confounded by differences in scale.<br>We therefore replaced the original subgroup comparisons with pooled unrestricted models in which respondent group was interacted with the opt-in ASC and all attribute coefficients. Overall respondent-group differences were assessed using a likelihood-ratio test for the conditional logit models and joint Wald and likelihood-ratio tests for the parsimonious RPL models.<br><br>None of the overall tests was statistically significant (conditional logit: LR $\chi^2(24)=34.10$, *p*=0.083; parsimonious RPL: Wald $\chi^2(24)=28.22$, *p*=0.251; LR $\chi^2(24)=32.79$, *p*=0.109). We therefore found no statistically significant overall evidence that preferences differed between peer workers and recipients. The full-sample parsimonious RPL was retained as the primary analysis, and subgroup-specific conclusions based on separate models were removed.<br><br>**Added text (Methods, lines 3332 - 338)**<br>"Formal comparisons between PSWs and PSRs were undertaken using a pooled unrestricted model in which the respondent group was interacted with the alternative-specific constant and all attribute coefficients. This approach permits direct statistical testing of subgroup differences while avoiding comparisons of coefficients from separately estimated models, which may be confounded by scale differences. Overall respondent-group differences were assessed using a likelihood-ratio test for the conditional logit models and joint Wald and likelihood-ratio tests for the parsimonious RPL models."<br><br>**Added text (Results, lines 416 - 421)**<br>"Formal subgroup comparisons were conducted using pooled interaction models. Neither the conditional logit nor the parsimonious RPL model showed significant overall preference

	differences between PSWs and PSRs (conditional logit: LR $\chi^2(24)=34.10$, $p=0.083$; parsimonious RPL: Wald χ^2 $(24)=28.22$, $p=0.251$; LR $\chi^2(24)=32.79$, $p=0.109$; Supplementary Table S4). The full-sample parsimonious RPL was therefore retained as the primary analysis (Table 3)."
The qualitative component is a nice, and seldomly explored in DCEs, contribution, but it feels disjointed from the preference results. I suggest the authors try to reflect on their preferences and try to pick out if there's any internal validity of the subgroup analysis results (which seems to be one of their main research questions) based on the qualitative data obtained. For example, are peer workers signalling a higher sensitivity to experience and integration. I appreciate there may be limited data, but this would significantly enhance the validity of the results and provide insights that can truly contribute to the literature.	We agree that the qualitative findings could be more effectively integrated with the discrete choice experiment results. Accordingly, we have substantially revised the Discussion so that the qualitative findings are now used to interpret and contextualise the quantitative preference estimates.<br><br>For example, participants consistently described experienced peer workers as being more credible, better able to navigate mental health services, and more capable of supporting recovery. These themes align closely with the strong quantitative preference for experienced peer workers observed across both econometric models. Similarly, participants frequently emphasised the importance of integration between peer-support services and primary care, describing continuity of care, communication with clinicians, and coordinated referrals as essential features of effective peer support. The qualitative findings help explain why continuity, communication and coordinated referral pathways were regarded as important, although the corresponding integration coefficients were not statistically significant in the primary parsimonious RPL model.<br><br>Importantly, because the formal interaction analyses did not demonstrate statistically significant subgroup differences, these qualitative findings are interpreted as explaining why particular programme characteristics were valued rather than validating statistically significant differences between peer workers and recipients. We believe this substantially strengthens the coherence of the mixed-methods findings.<br><br>**Added text (Discussion, lines 578 - 593)**

	"Respondents also described experienced PSWs as more credible, empathetic and capable of navigating complex mental health systems. These themes closely aligned with the quantitative finding that PSWs with greater experience were consistently preferred across both the conditional logit and parsimonious RPL models.<br><br>Similarly, respondents emphasised continuity of care, collaboration with clinicians and coordinated referral pathways as important components of effective peer support. These qualitative findings are directionally consistent with the positive preferences observed for integrated models of peer support, particularly those involving established contact persons or combined in-clinic and external service delivery.<br><br>Although some qualitative comments suggested that PSWs and PSRs may place different emphasis on particular aspects of peer support, the pooled interaction analyses did not demonstrate statistically significant overall differences in preferences between respondent groups. The qualitative findings therefore provide explanatory context for the overall preference patterns identified in the DCE rather than evidence of statistically significant subgroup differences."
The presentation of the task with the opt-out alternative levels each defined as "387 - " is unusual and may inadvertently be interpreted as indifference rather than zero part-worth utility, which could be problematic from a modelling perspective. I assume this was pretested in the pilot to ensure the opt-out was interpreted as intended. But I raise this	We thank the reviewer for identifying this inconsistency. It arose because Figure 1 inadvertently reflected an earlier iteration of the survey. In the final version administered to participants, the alternative was labelled "Opt-out (no peer support)". This has now been corrected in both the manuscript and Figure 1 to accurately reflect the survey instrument used in the study.

given the ASC's comment above. Can the authors reflect on this and incorporate to manuscript as appropriate.	

VERSION 2 - REVIEW

**Reviewer**	**1**
**Name**	**Hews-Girard, Julia**
**Affiliation**	**University of Calgary, Faculty of Nursing**
**Date**	**23-Jul-2026**
**COI**	

Thank you for your responses.

I have nothing further.

**Reviewer**	**2**
**Name**	**Loría-Rebolledo, Luis Enrique**
**Affiliation**	**University of Aberdeen**
**Date**	**05-Aug-2026**
**COI**	

Thank you for the authors for revising their paper. I think it is much improved and will contribute to the literature. Authors have addressed all my comments, although I have one further observation/comment to strengthen and one edit that needs addressing:

The new specification of the ASC as the opt in I think is more intuitive and better conveys their results. From this, the authors appear to want to make clear that the negative parameter is not reflective of respondents preferring the opt-out as they, correctly, note the confounding of the reference levels. Accordingly, they state in Lines 425: "This should not be interpreted as an overall preference for opting out: the opt-out alternative was selected in 50 of 672 choice tasks (7.4%), and 17 of 84 respondents selected it at least once (Supplementary Table S3)." I would suggest adding a statement to support this interpretation, something like "…as a peer support programme will have features (such as …) that will increase its utility above the opt-out's" to signal how part-worths from the

remaining attributes will likely push the deterministic component of a standard peer support programme into positive territory (as one would expect just by inspecting the estimates).

Authors report some negative standard deviations in Table 3 and in the Supplementary Material. Since SDs are non-negative by definition, this is likely reflecting how the random draws are generated in STATA (and most software): they should report as positive.

VERSION 2 - AUTHOR RESPONSE

Reviewer: 2
Dr. Luis Enrique Loría-Rebolledo, University of Aberdeen
Comments to the Author

Comments to the Author	Authors' responses to comments
Thank you for the authors for revising their paper. I think it is much improved and will contribute to the literature. Authors have addressed all my comments, although I have one further observation/comment to strengthen and one edit that needs addressing:<br>The new specification of the ASC as the opt in I think is more intuitive and better conveys their results. From this, the authors appear to want to make clear that the negative parameter is not reflective of respondents preferring the opt-out as they, correctly, note the confounding of the reference levels. Accordingly, they state in Lines 425: "This should not be interpreted as an overall preference for opting out: the opt-out alternative was selected in 50 of 672 choice tasks (7.4%), and 17 of 84 respondents selected it at least once (Supplementary Table S3)." I would suggest adding a statement to support this interpretation, something like "…as a peer support programme will have features (such as …) that will increase its utility above the opt-out's" to signal how part-worths from the remaining attributes will likely push the deterministic component of a standard peer support programme into positive territory (as one would expect just by inspecting the estimates).	Thank you for this helpful suggestion. We agree that the interpretation of the negative opt-in alternative-specific constant (ASC) can be strengthened by making explicit that the ASC represents the baseline utility of a hypothetical peer-support programme composed entirely of the reference attribute levels. The overall utility of a peer-support programme also incorporates the part-worth utilities associated with its attribute levels. We have therefore added text clarifying that positively valued programme characteristics can offset the negative baseline ASC and increase the utility of a peer-support programme relative to the opt-out alternative.<br><br>**Revised text (Results) – Pages 422 - 431:**<br>*"The negative opt-in alternative-specific constant indicated that a hypothetical peer-support programme composed entirely of the reference attribute levels had lower baseline utility than the opt-out alternative. This should not be interpreted as an overall preference for opting out: the opt-out alternative was selected in 50 of 672 choice tasks (7.4%), and 17 of 84 respondents selected it at least once (Supplementary Table S3). The utility of a peer-support programme also reflects the part-worth utilities associated with its attribute levels, such that positively valued characteristics, including community-based delivery, more frequent sessions, individual delivery and greater peer-worker experience, can offset the negative baseline ASC and increase programme utility relative to opting out."*

Comments to the Author	Authors' responses to comments
Authors report some negative standard deviations in Table 3 and in the Supplementary Material. Since SDs are non-negative by definition, this is likely reflecting how the random draws are generated in STATA (and most software): they should report as positive.	Thank you for this observation. We agree that standard deviations are non-negative by definition. In the previous revision, we retained the signs of the estimated random-parameter scale terms as produced by the estimation routine and explicitly noted in Table 3 and the Supplementary Material that the signs were not substantively meaningful and should be interpreted in absolute terms. We appreciate, however, that because these quantities are presented as standard deviations, reporting them as non-negative values is clearer and avoids potential ambiguity.<br><br>We have therefore removed the negative signs from the reported standard deviations in Table 3 and the Supplementary Material and now present all estimated random-parameter standard deviations as non-negative values (i.e., their absolute values). This is a presentational change only and does not affect the associated standard errors, statistical significance, model estimates, or interpretation of preference heterogeneity. We have also updated the relevant table notes to reflect this change.<br><br>**Revised table note for Tables 3, Supplementary Table S2 and Supplementary Table S4:**<br>*"Estimated standard deviations of the random parameters are reported as non-negative values. Statistically significant standard deviations indicate evidence of unobserved preference heterogeneity around the corresponding mean coefficients."*
