# Supplementary material for: Lived experience preferences for mental health peer support services in Australian primary care: a discrete choice experiment with qualitative analysis
Source: BMJ Open. 2026 Nov 9;16(11):e112132. doi: 10.1136/bmjopen-2025-112132 (PMC13756459; doi:10.1136/bmjopen-2025-112132)
Supplement: Supplementary file 2 [file bmjopen-16-11-s001.docx]

**SUPPLEMENTARY MATERIALS**

**Supplementary Table S1: Development of candidate attributes and selection of final Discrete Choice Experiment (DCE)**

| **Initial candidate attribute** | **Primary source(s)** | **Retained in final DCE?** | **Final form in the DCE** | **Notes on refinement** |
| --- | --- | --- | --- | --- |
| 1. Frequency of peer support sessions | Literature review (7,32); lived experience steering group | Yes | Monthly, bi-weekly, weekly, or on-demand | Retained with minor refinement of levels. |
| 2. Format of peer support | Literature review (7,32); stakeholder discussions | Yes | Online chat/forum, phone/video calls, individual sessions, or group sessions | Retained with simplified wording. |
| 3. Peer worker qualifications | Literature review (7,32,34); lived experience experts | Yes | Peer qualification, obtaining qualification, or lived experience only | Simplified following stakeholder feedback. |
| 4. Peer worker training duration | Literature review (34); research team | No | Replaced by peer worker experience | Practical experience was considered more meaningful than duration of formal training. |
| 5. Duration of individual peer support sessions | Literature review (7,32); stakeholder discussions | No | — | Excluded because programme duration was considered more relevant to service design. |
| 6. Location of peer support sessions | Literature review (7,32); stakeholder discussions | Yes | Primary care clinic, community, online, respondent’s home | Retained with simplified descriptions. |
| 7. Availability of peer support | Literature review (7,32); stakeholder discussions | No | — | Excluded to reduce respondent burden and because it was considered less actionable. |
| 8. Integration with primary care | Literature review (7,32); Department of Health evidence review (34); steering group | Yes | Offered within primary care clinic, external services, established contact person, combination model | Refined to reflect realistic models of primary care integration. |
| 9. Duration of peer support programme | Literature review (7,32); stakeholder discussions | Yes | 4 weeks, 8 weeks, 12 weeks, ongoing/open-ended | Retained unchanged. |
| 10. Identity, diagnosis and cultural background | Lived experience steering group; literature review (1,7) | Yes | Similar age, diagnosis, gender or culture | Refined from broader cultural sensitivity to peer service recipient (PSR) matching characteristics. |
| 11. Evaluation and feedback mechanisms | Literature review (7,32); research team | No | — | Considered an implementation feature rather than a service characteristic. |
| 12. Referral/access pathway | Literature review (32); stakeholder discussions | No | — | Considered to describe access to services rather than characteristics of peer support itself. |
| 13. Choice of peer worker | Lived experience steering group | No | — | Considered important but largely captured by the identity-matching attribute. |
| 14. Ability to change peer worker | Steering group discussions | No | — | Considered an organisational policy rather than a peer support attribute. |
| 15. Involvement of family/carers | Literature review (32); stakeholder discussions | No | — | Relevant only to some PSRs and therefore not universally applicable. |
| 16. Type of peer support provided (emotional, practical, social) | Literature review (1,7); lived experience experts | No | — | Considered fundamental to peer work rather than a feature respondents would meaningfully trade off. |

***References***

7.	Repper J, Carter T. A review of the literature on peer support in mental health services. J Ment Health. 2011;20(4):392-411.

32.	Lloyd-Evans B, Mayo-Wilson E, Harrison B, et al. A systematic review and meta-analysis of randomised controlled trials of peer support for people with severe mental illness. BMC Psychiatry. 2014;14:39.

34.	Lawn S, Deer-Price B, Mohammadi L, et al. Rapid review of international peer-reviewed literature, and targeted environmental scan of the grey literature, to identify innovative service delivery models to support increased engagement of the peer workforce. Adelaide: Lived Experience Australia Ltd; 2024.

**Survey background information, participant instructions and example choice set**

**Study Title: Investigating Stakeholders’ Preferences for the Design of a ‘best fit’ Peer Support Intervention for Primary Care for People with Long-term Mental Health Conditions.**

We invite you to participate in a research project that asks about your views and preferences for Peer Support Intervention for Primary Care for people with Long-term Mental Health Conditions.

The results from this survey will be helpful in guiding the future planning and delivery of mental health services in Australia.

Your participation in this survey is entirely voluntary. All of the information you provide will be confidential. It will be used for research purposes only and will not be used in any way in which you can be identified.

Thank you for your co-operation.

**Section B: Choosing Between Peer Support Packages**

In this section, we would like you to choose between two hypothetical peer support packages. You will need to compare the characteristics of each package and then choose the package that you would prefer at this point in time.

The following scenarios describe peer support packages for people with mental health conditions. The packages differ according to several characteristics, including the frequency of peer support sessions, the format of peer support, peer worker qualifications and experience, the location of peer support sessions, integration with primary care, the duration of the peer support programme, and the identity, diagnosis and cultural background of the peer worker.

You will be presented with **eight** **(8) pairs of hypothetical peer support packages**. For each choice situation, please imagine that **you, or someone you care for, has a mental health condition and requires peer support**. We ask you to choose the package that you would most prefer if you were offered these two options. If you would not choose either package, you may select the **"No peer support"** option.

The possible differences between the packages are described below.

**1. Frequency of peer support sessions**

- Weekly
- Bi-weekly
- Monthly
- On-demand (as needed)

**2. Format of peer support**

- Group sessions (small groups)
- Individual sessions (one-on-one)
- Online chat/forum
- Phone/video calls

**3. Peer worker qualifications**

- Peer worker with lived experience only
- Peer worker who has completed a recognised peer qualification
- Peer worker currently obtaining a recognised peer qualification

**4. Peer worker experience**

- Less than 6 months of providing peer support
- Between 6 and 12 months of providing peer support
- Between 12 and 24 months of providing peer support
- More than 24 months of providing peer support

**5. Location of peer support sessions**

- Primary care clinic
- Community location
- Online platform
- Respondent’s home

**6. Integration with primary care**

- Peer support offered within the primary care clinic
- Referral to external peer support services
- Established contact persons at the primary care clinic and peer support organisation to ensure continuity of communication
- Combination of in-clinic and external options

**7. Duration of the peer support programme**

- 4 weeks
- 8 weeks
- 12 weeks
- Ongoing/open-ended

**8. Identity, diagnosis and cultural background of the peer worker**

- Peer worker of a similar age
- Peer worker of a similar diagnosis
- Peer worker of a similar gender and/or sexual identity
- Peer worker of a similar cultural background

Eight choice tasks are presented below. Some of the characteristics of each package change from one choice task to the next. For each choice task, please indicate **which package you would choose**.

**Figure 1: Example Choice Set**

| **Attribute** | **Alternative A** | **Alternative B** | **Opt-out (No peer support)** |
| --- | --- | --- | --- |
| Frequency of peer support sessions | On-demand | Weekly | No peer support |
| Format of peer support | Individual sessions | Group sessions | No peer support |
| Peer worker qualifications | Lived experience only | Peer qualification | No peer support |
| Peer worker experience | Less than 6 months | 12–24 months | No peer support |
| Location of peer support sessions | Primary care clinic | Online | No peer support |
| Integration with primary care | Established contact person | Established contact person | No peer support |
| Duration of peer support programme | 8 weeks | 8 weeks | No peer support |
| Identity, diagnosis and cultural background of peer worker | Similar culture | Similar age | No peer support |

**Choice Question**

☐ I would choose **Alternative A**

☐ I would choose **Alternative B**

☐ I would choose the **Opt-out (No peer support)** option

**Supplementary Table S2. Conditional logit and parsimonious random-parameter logit estimates**

| **Attribute level** | **Conditional logit Coefficient (SE)** | **Parsimonious RPL Coefficient (SE)** |
| --- | --- | --- |
| **Alternative-specific constant** | | |
| Opt-in alternative-specific constant | -0.905 (0.463) | -1.604** (0.599) |
| **Peer worker qualifications** | | |
| Peer qualification | 0.566** (0.206) | 0.838** (0.295) |
| Obtaining a peer qualification | 0.248 (0.198) | 0.350 (0.264) |
| *Lived experience only* | *Reference* | *Reference* |
| **Peer worker experience of providing peer support** | | |
| 6–12 months | 0.552 (0.285) | 0.761* (0.328) |
| 12–24 months | 0.453 (0.284) | 0.554 (0.317) |
| More than 24 months | 0.885** (0.278) | 1.293** (0.407) |
| *Less than 6 months* | *Reference* | *Reference* |
| **Location of peer support sessions** | | |
| Primary care clinic | 0.408 (0.244) | 0.601 (0.326) |
| Community | 0.870*** (0.208) | 1.326*** (0.351) |
| Respondent's home | 0.520* (0.211) | 0.812* (0.340) |
| *Online* | *Reference* | *Reference* |
| **Peer support integration with primary care** | | |
| Referral to external services | 0.124 (0.213) | 0.250 (0.294) |
| Established contact person | 0.175 (0.234) | 0.280 (0.352) |
| Combined in-clinic and external services | 0.426* (0.188) | 0.550 (0.288) |
| *Within primary care clinic* | *Reference* | *Reference* |
| **Duration of peer support programme** | | |
| 8 weeks | 0.495* (0.241) | 0.503 (0.332) |
| 12 weeks | 0.749** (0.270) | 0.866* (0.358) |
| Ongoing/open-ended | 0.987** (0.314) | 1.281** (0.462) |
| *4 weeks* | *Reference* | *Reference* |
| **Identity, diagnosis and cultural background of the peer support provider** | | |
| Similar age | -0.039 (0.208) | 0.008 (0.315) |
| Similar gender | -0.064 (0.236) | 0.005 (0.299) |
| Similar cultural background | 0.085 (0.176) | 0.094 (0.244) |
| *Similar diagnosis* | *Reference* | *Reference* |
| **Frequency of peer support sessions** | | |
| Weekly | 0.829** (0.289) | 1.359** (0.461) |
| Bi-weekly | 0.432** (0.165) | 0.737** (0.279) |
| On-demand | 0.542** (0.208) | 0.816* (0.369) |
| *Monthly* | *Reference* | *Reference* |
| **Format of peer support** | | |
| Individual sessions | 0.678*** (0.166) | 1.037*** (0.273) |
| Online chat/forum | 0.056 (0.311) | 0.115 (0.490) |
| Phone/video calls | 1.191 (0.611) | 1.541* (0.677) |
| *Group sessions* | *Reference* | *Reference* |
| **Standard deviations of random parameters** | | |
| Peer worker qualifications: peer qualification |  | 0.554 (0.449) |
| Peer worker experience of providing peer support: more than 24 months |  | 1.014* (0.424) |
| Location of peer support sessions: community |  | 0.778 (0.458) |
| Location of peer support sessions: respondent’s home |  | 1.140** (0.373) |
| Peer support integration with primary care: established contact person |  | 1.232*** (0.345) |
| Peer support integration with primary care: combined in-clinic and external services |  | 0.463 (0.578) |
| Duration of peer support programme: ongoing/open-ended |  | 1.244* (0.556) |
| Frequency of peer support sessions: weekly |  | 0.423 (0.673) |
| Frequency of peer support sessions: on-demand |  | 1.064** (0.403) |
| Format of peer support: individual sessions |  | 1.002*** (0.266) |
| Format of peer support: online chat/forum |  | 1.191 (1.125) |
| **Model statistics** | | |
| Observations | 2,016 | 2,016 |
| Respondents | 84 | 84 |
| Log likelihood | -535.970 | -515.013 |
| Akaike information criterion | 1,119.940 | 1,100.025 |
| Bayesian information criterion | 1,254.553 | 1,296.336 |

Abbreviations: AIC, Akaike information criterion; ASC, alternative-specific constant; BIC, Bayesian information criterion; RPL, random-parameter logit; SE, standard error.

Notes: Coefficients are presented with standard errors in parentheses. Reference levels are shown as the final row within each attribute group. The parsimonious RPL model included 11 normally distributed random coefficients and was estimated by maximum simulated likelihood using 500 scrambled Halton draws. Estimated standard deviations of the random parameters are reported as non-negative values. Statistically significant standard deviations indicate evidence of unobserved preference heterogeneity around the corresponding mean coefficients. * p < 0.05, ** p < 0.01, *** p < 0.001.

**Supplementary Table S3. Observed Opt-out (No peer support) behaviour**

| **Measure** | **Value** |
| --- | --- |
| Respondents | 84 |
| Completed choice tasks | 672 |
| Opt-out choices | 50 (7.4%) |
| Respondents selecting opt-out at least once | 17 (20.2%) |
| Respondents never selecting opt-out | 67 (79.8%) |
| Respondents selecting Opt-out (No peer support) in all eight tasks | 1 (1.2%) |

The Opt-out (No peer support) alternative represented selecting neither of the two hypothetical peer-support programmes.

**Supplementary Table S4. Sensitivity analyses of alternative-specific constant specifications and respondent-group preference differences using conditional logit and parsimonious random-parameter logit models.**

| **Variable** | **CLOGIT: Two ASCs** | **CLOGIT: Respondent-group interactions** | **Parsimonious RPL: Two ASCs** | **Parsimonious RPL: Respondent-group interactions** |
| --- | --- | --- | --- | --- |
| **Panel A. Mean preference coefficients** |  |  |  |  |
|  |  |  |  |  |
| **Alternative-specific constants** | |  |  |  |
| Alternative 1 ASC | -0.919* (0.457) |  | -1.661** (0.620) |  |
| Alternative 2 ASC | -0.901 (0.469) |  | -1.594** (0.599) |  |
| Common opt-in ASC |  | -0.063 (0.695) |  | -0.446 (0.809) |
| **Qualifications** |  |  |  |  |
| Peer qualification | 0.576* (0.227) | 0.403 (0.327) | 0.876** (0.314) | 0.546 (0.412) |
| Obtaining peer qualification | 0.254 (0.208) | 0.206 (0.298) | 0.370 (0.270) | 0.239 (0.372) |
| *Lived experience only* | *Reference* | *Reference* | *Reference* | *Reference* |
| **Experience** |  |  |  |  |
| 6–12 months | 0.540 (0.297) | 0.757 (0.417) | 0.719* (0.347) | 1.052* (0.467) |
| 12–24 months | 0.441 (0.307) | 0.664 (0.398) | 0.509 (0.341) | 0.944* (0.458) |
| More than 24 months | 0.874** (0.289) | 0.875* (0.401) | 1.261** (0.416) | 1.306* (0.586) |
| *Less than 6 months* | *Reference* | *Reference* | *Reference* | *Reference* |
| **Location** |  |  |  |  |
| Primary care clinic | 0.412 (0.246) | 0.289 (0.313) | 0.617 (0.329) | 0.377 (0.449) |
| Community | 0.873*** (0.210) | 0.830** (0.295) | 1.342*** (0.354) | 1.204** (0.460) |
| Respondent's home | 0.516* (0.212) | 0.315 (0.278) | 0.801* (0.340) | 0.500 (0.463) |
| *Online* | *Reference* | *Reference* | *Reference* | *Reference* |
| **Integration with primary care** |  |  |  |  |
| External referral | 0.118 (0.217) | 0.241 (0.278) | 0.232 (0.297) | 0.384 (0.413) |
| Established contact person | 0.176 (0.234) | 0.549 (0.325) | 0.289 (0.353) | 0.701 (0.504) |
| Combined in-clinic and external | 0.429* (0.188) | 0.776** (0.264) | 0.561 (0.288) | 0.971* (0.415) |
| *Within primary care clinic* | *Reference* | *Reference* | *Reference* | *Reference* |
| **Duration** |  |  |  |  |
| 8 weeks | 0.503* (0.239) | 0.138 (0.317) | 0.533 (0.342) | 0.056 (0.456) |
| 12 weeks | 0.756** (0.263) | 0.171 (0.333) | 0.896* (0.366) | 0.011 (0.498) |
| Ongoing/open-ended | 1.005*** (0.296) | 0.984* (0.437) | 1.347** (0.497) | 1.230 (0.646) |
| *4 weeks* | *Reference* | *Reference* | *Reference* | *Reference* |
| **Peer worker background** |  |  |  |  |
| Similar age | -0.039 (0.209) | -0.371 (0.306) | 0.004 (0.316) | -0.562 (0.449) |
| Similar gender | -0.065 (0.235) | -0.207 (0.320) | -0.004 (0.300) | -0.132 (0.404) |
| Similar cultural background | 0.088 (0.177) | 0.041 (0.247) | 0.102 (0.245) | 0.000 (0.341) |
| *Similar diagnosis* | *Reference* | *Reference* | *Reference* | *Reference* |
| **Frequency** |  |  |  |  |
| Weekly | 0.829** (0.289) | 0.833 (0.468) | 1.368** (0.462) | 1.378* (0.652) |
| Bi-weekly | 0.429* (0.169) | 0.322 (0.252) | 0.728** (0.280) | 0.715 (0.403) |
| On-demand | 0.537* (0.209) | 0.651 (0.343) | 0.808* (0.369) | 0.969 (0.541) |
| *Monthly* | *Reference* | *Reference* | *Reference* | *Reference* |
| **Format** |  |  |  |  |
| Individual sessions | 0.684*** (0.167) | 0.388 (0.246) | 1.058*** (0.279) | 0.746 (0.391) |
| Online chat/forum | 0.073 (0.340) | -0.178 (0.443) | 0.172 (0.514) | -0.096 (0.722) |
| Phone/video calls | 1.180 (0.620) | 1.455 (0.820) | 1.509* (0.679) | 1.845 (1.053) |
| *Group sessions* | *Reference* | *Reference* | *Reference* | *Reference* |
| **Panel B. Peer Service Recipient (PSR) × attribute interaction terms** |  |  |  |  |
| PSR × Opt-in ASC |  | -1.948* (0.968) |  | -2.628* (1.202) |
| PSR × Peer qualification |  | 0.300 (0.419) |  | 0.477 (0.578) |
| PSR × Obtaining peer qualification |  | 0.125 (0.389) |  | 0.281 (0.539) |
| PSR × Experience: 6–12 months |  | -0.416 (0.584) |  | -0.645 (0.666) |
| PSR × Experience: 12–24 months |  | -0.381 (0.590) |  | -0.724 (0.644) |
| PSR × Experience: more than 24 months |  | 0.089 (0.587) |  | 0.010 (0.816) |
| PSR × Location: primary care clinic |  | 0.404 (0.480) |  | 0.665 (0.685) |
| PSR × Location: community |  | 0.137 (0.405) |  | 0.238 (0.647) |
| PSR × Location: respondent’s home |  | 0.340 (0.424) |  | 0.539 (0.656) |
| PSR × Integration: external referral |  | -0.189 (0.412) |  | -0.214 (0.581) |
| PSR × Integration: established contact person |  | -0.715 (0.486) |  | -0.879 (0.717) |
| PSR × Integration: combined in-clinic and external |  | -0.604 (0.386) |  | -0.764 (0.555) |
| PSR × Duration: 8 weeks |  | 0.860 (0.486) |  | 1.131 (0.700) |
| PSR × Duration: 12 weeks |  | 1.355* (0.535) |  | 1.947* (0.759) |
| PSR × Duration: ongoing/open-ended |  | 0.249 (0.659) |  | 0.340 (0.928) |
| PSR × Background: similar age |  | 0.690 (0.458) |  | 1.155 (0.667) |
| PSR × Background: similar gender |  | 0.399 (0.474) |  | 0.419 (0.629) |
| PSR × Background: similar cultural background |  | 0.174 (0.386) |  | 0.298 (0.508) |
| PSR × Frequency: weekly |  | -0.040 (0.595) |  | -0.105 (0.872) |
| PSR × Frequency: bi-weekly |  | 0.203 (0.347) |  | 0.104 (0.554) |
| PSR × Frequency: on-demand |  | -0.171 (0.434) |  | -0.205 (0.741) |
| PSR × Format: individual sessions |  | 0.623 (0.350) |  | 0.722 (0.534) |
| PSR × Format: online chat/forum |  | 0.456 (0.641) |  | 0.384 (1.000) |
| PSR × Format: phone/video calls |  | -0.521 (1.204) |  | -0.412 (1.383) |
| **Panel C. Estimated standard deviations of random parameters (RPL models only)** |  |  |  |  |
| Peer qualification |  |  | 0.552 (0.451) | 0.459 (0.544) |
| Experience: more than 24 months |  |  | 1.013* (0.426) | 1.191** (0.422) |
| Location: community |  |  | 0.777 (0.454) | 0.652 (0.546) |
| Location: respondent’s home |  |  | 1.126** (0.376) | 0.999* (0.393) |
| Integration: established contact person |  |  | 1.235*** (0.346) | 1.200*** (0.341) |
| Integration: combined in-clinic and external |  |  | 0.459 (0.582) | 0.445 (0.601) |
| Duration: ongoing/open-ended |  |  | 1.254* (0.557) | 1.084 (0.575) |
| Frequency: weekly |  |  | 0.437 (0.668) | 0.300 (0.830) |
| Frequency: on-demand |  |  | 1.060** (0.402) | 1.177** (0.406) |
| Format: individual sessions |  |  | 1.012*** (0.269) | 1.042*** (0.275) |
| Format: online chat/forum |  |  | 1.178 (1.115) | 1.415 (1.061) |
| **Model statistics** |  |  |  |  |
| Observations | 2,016 | 2,016 | 2,016 | 2,016 |
| Log likelihood | -535.962 | -518.918 | -514.946 | -498.620 |
| AIC | 1,121.923 | 1,133.836 | 1,101.892 | 1,115.239 |
| BIC | 1,262.145 | 1,403.062 | 1,303.812 | 1,446.163 |
|  |  |  |  |  |
|  |  |  |  |  |
|  |  |  |  |  |
| Equality test^a^: Alternative 1 ASC = Alternative 2 ASC | χ²(1)=0.02;  p=0.879 |  | χ²(1)=0.13; p=0.716 |  |
|  |  |  |  |  |
| Overall test of respondent-group differences^b^ |  | LR χ²(24)=34.10; p=0.083 |  | Wald χ²(24)=28.22; p=0.251; LR χ²(24)=32.79; p=0.109 |

**Abbreviations:** AIC, Akaike information criterion; ASC, alternative-specific constant; BIC, Bayesian information criterion; CLOGIT, conditional logit; RPL, random-parameter logit.

**Notes:** Coefficients are presented with standard errors in parentheses. In the respondent-group interaction models, peer support workers (PSWs) are the reference group; therefore, the main coefficients represent preferences among PSWs, while the interaction terms represent differences in preferences among peer service recipients (PSRs) relative to PSWs. In the two-ASC models, separate constants were estimated for Alternatives 1 and 2, with the opt-out (no peer support) alternative as the reference. In the interaction models, a common opt-in ASC was estimated relative to the opt-out alternative. The parsimonious RPL models included 11 normally distributed random coefficients and were estimated by maximum simulated likelihood using 500 scrambled Halton draws. Panel C reports the estimated standard deviations of the random coefficients. Standard deviations that were statistically significantly different from zero indicate evidence of unobserved preference heterogeneity around the corresponding mean coefficients. Estimated standard deviations of the random parameters are reported as non-negative values. Statistically significant standard deviations indicate evidence of unobserved preference heterogeneity around the corresponding mean coefficients. Reference attribute levels are presented as the final row within each attribute group. Lower AIC and BIC values indicate better model fit after accounting for model complexity.

ᵃ The equality tests showed that the ASCs for Alternatives 1 and 2 did not differ significantly in either model (conditional logit: χ²(1)=0.02, p=0.879; parsimonious RPL: χ²(1)=0.13, p=0.716), supporting the use of a single common opt-in ASC.

ᵇ The overall tests assessed whether the opt-in ASC and all attribute coefficients differed jointly between PSWs and PSRs. No statistically significant overall respondent-group differences were identified in either the conditional logit model (LR χ²(24)=34.10, p=0.083) or the parsimonious RPL model (Wald χ²(24)=28.22, p=0.251; LR χ²(24)=32.79, p=0.109). Although some individual interaction coefficients were statistically significant, these findings should be interpreted as exploratory given the non-significant overall joint tests. The full-sample parsimonious RPL model was therefore retained as the primary model.

*p<0.05; **p<0.01; ***p<0.001.
