# Supplementary material for: Lived experience preferences for mental health peer support services in Australian primary care: a discrete choice experiment with qualitative analysis
Source: BMJ Open. 2026 Nov 9;16(11):e112132. doi: 10.1136/bmjopen-2025-112132 (PMC13756459; doi:10.1136/bmjopen-2025-112132)
Supplement: Supplementary file 3 [file bmjopen-16-11-s002.docx]

**DIRECT Checklist**

From: Ride J, Goranitis I, Meng Y, LaBond C, Lancsar E. A reporting checklist for discrete choice experiments in health: The DIRECT Checklist. *Pharmacoeconomics*. 2024;42(10):1161-1175. doi:10.1007/s40273-024-01431-6

| **Section**  **Item** | | **Page and paragraph** |
| --- | --- | --- |
| **Purpose and rationale** | |  |
| 1 | Describe the real-world context and decision-maker that the hypothetical choice context seeks to replicate or inform | Lines 78-87 |
| 2 | Provide a rationale for using a DCE to answer the research question | Lines 87-112 |
| **Attributes and levels^a^** | |  |
| 3 | Describe how attributes and levels were derived (e.g. literature review, interviews, focus groups, expert input) | Lines 141-244  Lines 267-292 |
| 4 | Provide the final list of attributes and levels | Lines 173-181 |
| **Experimental design** | |  |
| 5 | Report the number of alternatives per choice set and whether they were labelled or unlabelled | Figure 1  Lines 201-209 |
| 6 | Describe response options (e.g. forced choice, opt-out, status quo) | Lines 201-203 |
| 7 | Describe the type of experimental design (e.g. orthogonal, D-efficient, Bayesian efficient, partial profile) | Lines 232-234 |
| 8 | Describe which effects are identified in the design (e.g. main effects, higher order interactions, functional form) | Lines 234-236 |
| 9 | Describe the number of choice sets, blocks and choice sets per block | Lines 236-237 |
| 10 | Indicate how the experimental design was obtained (software, catalogue, other) | Lines 232-234 |
| **Survey design** | |  |
| 11 | Provide a sample choice set and the instructions and background information given to respondents (e.g. providing the survey as an appendix) | Figure 1  Supplementary information |
| 12 | Report any randomisation (e.g. choice set order, attribute order, alternative order, framing effects) | Lines 236-237 |
| 13 | Describe what was checked in piloting (e.g. understanding, respondent burden, timing, wording) | Lines 190-228 |
| 14 | Report whether information from the pilot was used to update the experimental design (e.g. priors, functional form of attributes) or survey design | Lines 211-228 |
| **Sample and data collection** | |  |
| 15 | Report respondent inclusion/exclusion criteria | Lines 247-260 |
| 16 | Describe how data were collected (e.g. mail, personal interview, web survey) | Lines 247-260 |
| 17 | Report the response rate or cooperation rate, if possible | Lines 367-368 |
| 18 | Report the final sample size and how the sample size was determined | Lines 367-368 |
| 19 | Describe respondent characteristics and representativeness of target population, if known | Lines 367-368  Table 1  Supplementary information |
| **Econometric analysis** | |  |
| 20 | Indicate coding of data (e.g. effects, dummy, continuous) including definitions | Lines 298-349  Table 3 |
| 21 | Report whether any respondents were removed and why (e.g. suspected fraudulent responses, rationality tests) | N/A |
| 22 | Provide the rationale for model choice (e.g. conditional logit, mixed logit, latent class) and assumptions (e.g. error variance) | Lines 298-349 |
| 23 | Report model specification | Lines 298-331 |
| **Reporting of results** | |  |
| 24 | Report the model performance, goodness of fit (if comparing models) | Lines 408-421 |
| 25 | Describe methods used for analysis of model results (e.g. calculation of marginal rate of substitution, attribute relative importance, welfare gain) | Lines 408-427  Table 3 |
| 26 | Report measures of precision for the output(s) of interest (e.g. confidence intervals) and how these were derived | Table 3 |
| ^a^For reporting of formative qualitative analysis, see <https://www.equator-network.org/reporting-guidelines/using-qualitative-methods-for-attribute-development-for-discrete-choice-experiments-issues-and-recommendations/> | | |
